# Integrated machine learning forecasting and grey wolf optimization for optimal operation of virtual power plants in smart distribution networks

**DOI:** 10.1038/s41598-026-65123-1

**Published:** 2026-08-08

**Authors:** Amal M. Abd El Hamid, Hebatallah H. Elzohri, Khairy Sayed

**Affiliations:** 1 Electricity Department, Faculty of Technology and Education, Sohag University, Sohag, 82524 Egypt; 2 Department of Electrical Engineering, College of Engineering, Sohag University, Sohag, Egypt

**Keywords:** Virtual power plants (VPP), Artificial neural networks, Distributed generation (DG), Power loss reduction, Voltage profile enhancement, ML, Load forecasting, Energy science and technology, Engineering, Mathematics and computing

## Abstract

The increasing penetration of distributed energy resources (DERs) necessitates intelligent coordination strategies that simultaneously enhance the technical, economic, and environmental performance of modern distribution networks. This paper proposes an integrated Virtual Power Plant (VPP) framework that combines optimal distributed generation (DG) planning, machine learning-based load forecasting, and battery energy storage system (BESS) scheduling within a unified energy management architecture. The proposed framework integrates the Grey Wolf Optimizer (GWO) for optimal DG placement and sizing with a hybrid Artificial Neural Network–Support Vector Machine (ANN–SVM) forecasting model to enable data-driven operational scheduling under varying load conditions. The methodology is validated using the IEEE 69-bus radial distribution system through comprehensive steady-state and time-series simulations. The optimization results demonstrate that the proposed planning strategy reduces active power losses by **61.7%** while improving the minimum bus voltage from **0.909 p.u.** to **0.966 p.u.**, thereby enhancing network voltage stability. The hybrid ANN–SVM forecasting model provides accurate hourly load predictions that support reliable energy scheduling and coordinated DER operation. Time-series simulations further verify that the coordinated dispatch of renewable energy resources and BESS maintains secure voltage profiles and stable system operation during peak-demand periods. Renewable generation supplies approximately **4.2 MWh/day**, while the BESS performs effective energy arbitrage that increases renewable energy utilization and reduces dependence on grid electricity. Comprehensive techno-economic evaluation confirms the practicality and scalability of the proposed VPP framework, achieving daily operating cost savings of **$1**,**741.32**, a discounted payback period of **3.8 years**, and an internal rate of return (IRR) of **30%**. Furthermore, increased renewable energy penetration leads to significant reductions in CO₂ emissions, demonstrating the environmental benefits of the proposed approach. The obtained results consistently demonstrate that the coordinated integration of metaheuristic optimization, hybrid machine learning forecasting, and intelligent energy storage management provides a robust, reproducible, and scalable solution for improving the reliability, operational efficiency, economic viability, and sustainability of future smart distribution networks.

## Introduction

The escalating integration of Distributed Energy Resources (DERs), encompassing Photovoltaic (PV) systems, Wind Turbines (WTs), and Battery Energy Storage Systems (BESS), is fundamentally transforming the operational paradigms of contemporary distribution networks. By employing a unified coordination layer, these heterogeneous DERs are aggregated into a Virtual Power Plant (VPP) architecture^1^. This framework facilitates the delivery of critical system-level services, including energy arbitrage, peak shaving, voltage regulation, and minimization of network losses^2^. In contrast to conventional utility-centric control methodologies, the VPP framework utilizes real-time forecasting and stochastic optimization to manage DER scheduling amidst dynamic pricing structures and the inherent volatility of renewable generation. Consequently, this coordinated approach enhances the technical stability, economic efficiency, and environmental sustainability of the electrical grid, positioning the VPP as a pivotal element in the transition toward decentralized energy management^3,4^.

Despite significant advancements, the deployment of distribution-level Virtual Power Plants (VPPs) remains constrained by three fundamental challenges^5^. Recent studies have employed machine-learning algorithms for optimal DG placement, demonstrating significant improvements in loss reduction and voltage profile^6,7^ Hybrid approaches combining neural networks with metaheuristic optimization, such as GWO, have shown promising results for load distribution forecasting in VPPs^6,7^ Comprehensive reviews on VPP optimization models have categorized various methodologies and highlighted emerging trends^8^, while recent advancements have focused on enhancing grid flexibility through coordinated DER management^9^. First, the optimal siting and sizing of Distributed Generation (DG) within radial feeders constitutes a complex, non-linear optimization problem due to the inherent coupling between voltage deviations, branch currents, and active/reactive power flows. Sub-optimal allocation frequently results in compromised voltage regulation and exacerbated network losses^10,11^. Second, the short-term forecasting of feeder demand, particularly when subjected to pronounced diurnal variability, is a critical determinant of operational scheduling. Inaccurate load predictions propagate through the system, leading to sub-optimal dispatch strategies and a subsequent degradation of grid reliability^12^. Third, the coordinated management of BESS with intermittent Photovoltaic (PV) and Wind Turbine (WT) generation, alongside time-varying electricity pricing, necessitates computationally tractable yet robust control policies. Achieving such coordination is particularly demanding when integrated with stringent distribution power-flow constraints, requiring a balance between algorithmic efficiency and practical efficacy^13^.

The global transition toward sustainable and decentralized power systems has necessitated the large-scale integration of Distributed Renewable Energy Sources (RES), specifically Photovoltaic (PV) and Wind Generation, into modern distribution networks^14^. Nonetheless, the inherent stochastic nature and intermittency of these resources introduce significant complexities regarding grid reliability, the minimization of operational expenditures, and the maintenance of voltage profiles. To address these technical and economic challenges, the Virtual Power Plant (VPP) has emerged as a robust operational paradigm. The VPP architecture enables the aggregation of heterogeneous Distributed Energy Resources (DERs), including Distributed Generation (DG), Battery Energy Storage Systems (BESS), and demand-side flexibility into a singular, coordinated entity. This coordinated framework facilitates active participation in wholesale energy markets and the provision of critical ancillary grid services, thereby optimizing the synergy between volatile generation and network demand^6,7^.

## VPP management framework: methodology and literature review

**Fig. 1:**
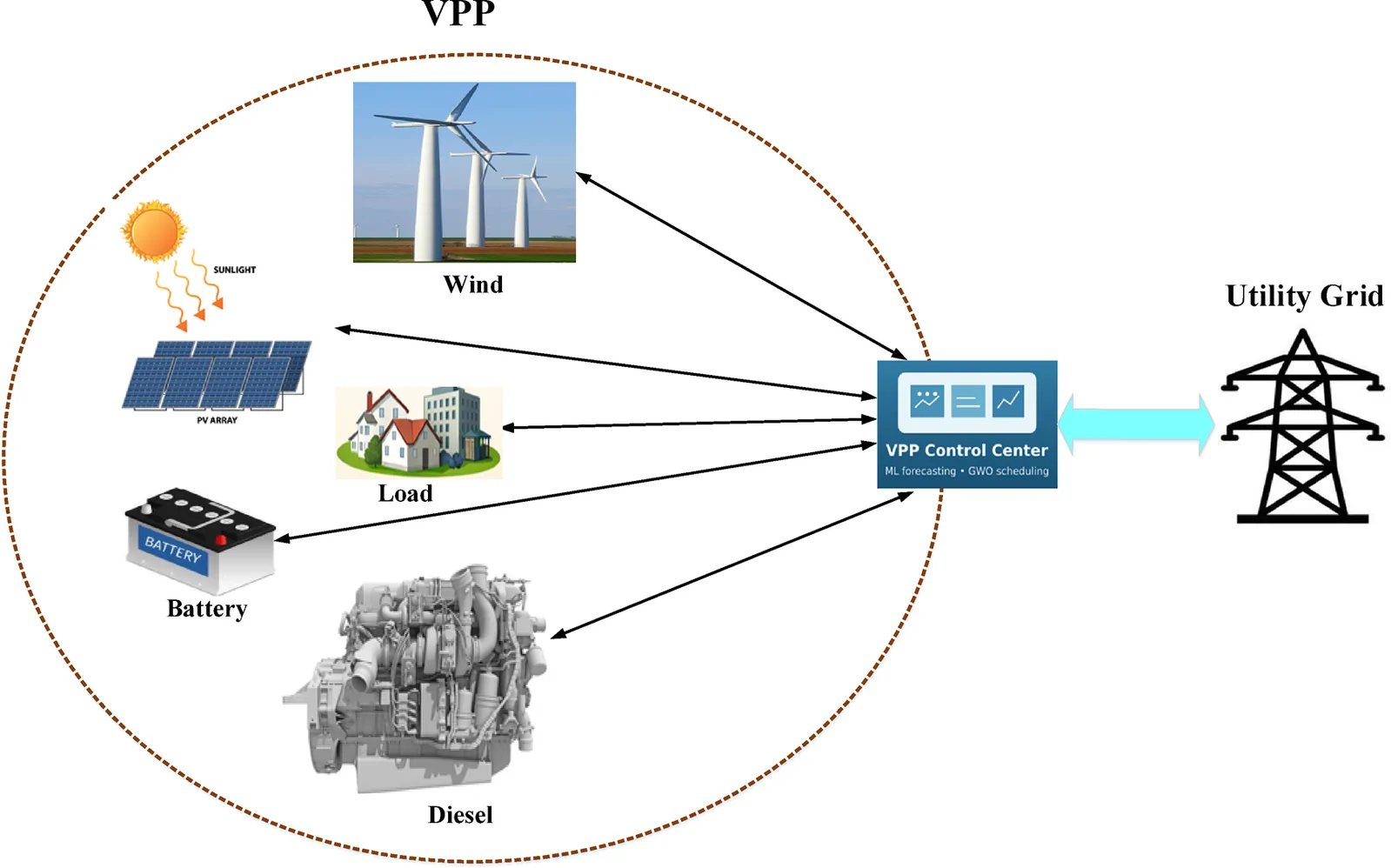
The architectural configuration of a Virtual Power Plant (VPP) integrated into a utility grid.

This research aims to bridge existing gaps in the literature by developing an integrated management framework that synthesizes neural network-based forecasting with Grey Wolf Optimization (GWO). This integration is designed to enhance the technical, economic, and environmental performance of Virtual Power Plants (VPPs) operating under realistic distribution network constraints^15^. The conceptual architecture of the Virtual Power Plant (VPP) and the integration of its constituent components within a conventional utility grid are illustrated in Fig. 1^16,17^. Recent advancements in Artificial Intelligence (AI), particularly deep learning (DL), offer sophisticated methodologies for addressing the complexities of VPP operational optimization. Due to their capacity to model intricate nonlinear relationships, DL architectures are uniquely suited for approximating the behavior of Distributed Generators (DGs) across diverse operating conditions^8^. By coupling deep learning with metaheuristic techniques such as Genetic Algorithms (GA), contemporary research is identifying increasingly flexible and scalable solutions for energy resource management and grid stability^9^. High-fidelity forecasting is the cornerstone of reliable VPP operations^18^. Uncertainties inherent in renewable generation and demand require robust forecasting models, as extensively reviewed in recent literature^19^. Deep learning techniques have emerged as powerful tools for short-term load forecasting, with comprehensive surveys detailing their applications and performance^20^.Furthermore, metaheuristic algorithms such as Golden Jackal Optimization have been successfully applied to the optimal allocation of DG and EV parking lots^21^. Uncertainties inherent in solar irradiance, wind speed, and electricity demand can propagate through the system, leading to significant dispatch errors, increased imbalance costs, and compromised system reliability. Consequently, the development of robust and precise predictive models has become a critical research priority within the domain of VPP management^19^.

A substantial body of literature demonstrates that machine learning (ML) and deep learning (DL) techniques consistently outperform traditional statistical baselines for short‑term forecasting of load and renewable energy outputs in VPP contexts^22^. Comprehensive reviews highlight the superior performance of ensemble learning, recurrent neural networks, convolutional architectures, and hybrid deep learning models when applied to forecasting tasks such as load prediction, renewable energy source (RES) output estimation, and price forecasting within smart‑grid and VPP environments. These findings are well documented in recent surveys on ML‑based forecasting for VPPs and deep learning‑based load prediction^20^. Within this context, the Levenberg-Marquardt (LM) training algorithm remains a favored approach for feedforward networks due to its rapid and stable convergence when optimizing nonlinear objectives, a vital attribute for systems requiring frequent model retraining^23^.

Even with optimized forecasts, the siting, sizing, and scheduling of Distributed Energy Resources (DERs) constitute a non-convex, multi-objective optimization problem characterized by nonlinear constraints and conflicting goals^21^. Under such conditions, gradient-based methods often prove inadequate, whereas metaheuristic algorithms are preferred for their derivative-free search capabilities and their ability to approximate global optima in high-dimensional spaces^24^. Among these, the Grey Wolf Optimizer (GWO), inspired by the social hierarchy and cooperative hunting mechanisms of wolves, has demonstrated competitive performance and rapid convergence on diverse engineering benchmarks^24,25^. Artificial intelligence technologies are increasingly being integrated into VPP management frameworks, as reviewed in recent studies^1,26^. AI-enhanced multi-timescale optimization strategies have been proposed to improve VPP operational efficiency^27^. Hybrid GWO-PSO approaches have demonstrated superior performance in optimal PV-DG placement and sizing^28^.In the context of distribution system operations, GWO has been successfully applied to the optimal placement and sizing of multiple DG units on standard feeders (notably the IEEE 69-bus system), yielding significant reductions in active power losses and marked improvements in voltage profiles^29–32^.

State-of-the-art VPP research increasingly shifts toward hybrid, end-to-end frameworks that fuse predictive ML/DL models with metaheuristic optimization. These architectures leverage forecasts to mitigate uncertainty while using optimization engines to translate those predictions into feasible operational schedules^1,26^. Contemporary research has increasingly utilized coupled architectures that integrate advanced forecasting models such as neural networks with attention mechanisms or ensemble learners with model predictive control (MPC) and metaheuristic dispatch engines. These integrated frameworks have demonstrated measurable improvements in operational expenditures, system flexibility, and robustness against the inherent volatility of Renewable Energy Sources (RES) when validated on realistic datasets^27^. Specifically, within distribution-level analyses, hybrid schemes that augment the Grey Wolf Optimizer (GWO) or its enhanced variants with machine learning components for tasks such as hyper parameter tuning, constraint handling, or surrogate modeling have consistently outperformed traditional single-technique baselines. These hybrid approaches facilitate superior performance on standard feeders by significantly reducing active power losses and enhancing voltage regulation^28^.

**Fig. 2:**
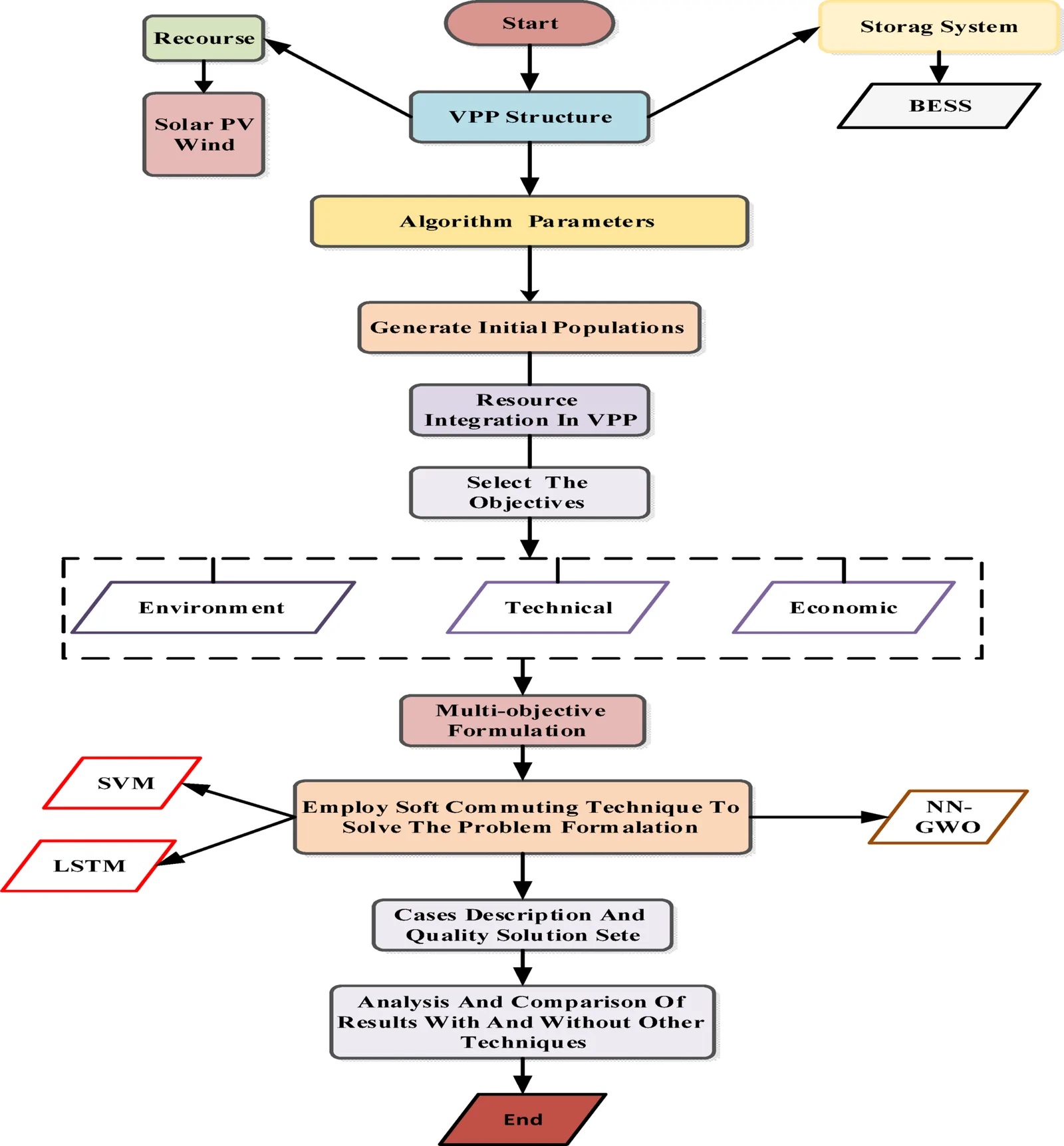
Flow Chart: operational sequence of the proposed methodological framework.

Furthermore, modern Virtual Power Plant (VPP) management paradigms have transcended purely technical Key Performance Indicators (KPIs) to prioritize market profitability and decarbonisation. Consequently, scholarly efforts have pivoted toward multi-market participation, encompassing energy arbitrage, reserve markets, Renewable Energy Certificates (RECs), and carbon emissions trading^33^. Research employing stochastic, robust, and game-theoretic formulations indicates that the explicit integration of carbon taxation, emission constraints, and multi-market arbitrage opportunities into VPP decision-making frameworks can fundamentally alter optimal dispatch strategies^34^. Such comprehensive modeling simultaneously scales revenue streams and mitigates environmental impacts within both isolated and multi-VPP operational contexts^35–37^. The operational sequence and algorithmic logic of the proposed methodological framework are depicted in Fig. 2. This schematic illustrates the hierarchical integration of the research objectives, the data-driven forecasting phase, and the multi-objective optimization procedure employed to derive the optimal system configuration^38^.

### Study motivation

Attaining peak Virtual Power Plant (VPP) performance necessitates the integration of high-fidelity forecasting models and robust multi-objective optimization frameworks capable of functioning within realistic distribution-network constraints. Much of the inherent technical complexity stems from the structural characteristics of actual distribution feeders, most notably radial topologies such as the IEEE 69-bus test system^38^. This benchmark is extensively utilized in the literature due to its high sensitivity to Distributed Generation (DG) placement and its accurate representation of practical distribution environments. Extant research employing the Grey Wolf Optimizer (GWO) for DG planning within the 69-bus architecture has consistently demonstrated substantial reductions in active power losses and marked enhancements in voltage stability. These findings corroborate the suitability of this specific network for the evaluation of advanced optimization frameworks and the validation of VPP-centric operational strategies^25^.

Furthermore, contemporary research leveraging machine-learning-based DG allocation continues to employ the IEEE 69-bus system in conjunction with other benchmark feeders, such as the IEEE 33-bus system^39^. This trend reinforces the system’s status as a standardized platform for the rigorous assessment of optimization and forecasting methodologies within high-complexity, realistic distribution networks. The persistent utilization of these benchmarks in recent literature facilitates the comparative validation of novel algorithmic frameworks, ensuring that performance improvements in voltage regulation and loss reduction are evaluated against established state-of-the-art results. Consequently, the 69-bus system remains an indispensable model for simulating the integration of distributed energy resources (DERs) and validating the scalability of proposed Virtual Power Plant (VPP) architectures^6,40^.

However, a review of the current literature reveals that few studies have integrated high fidelity neural network-based forecasting with GWO-driven multi-objective optimization within a singular, unified framework for VPP management on realistic radial networks. While individual components of optimization and prediction have been explored, the synergistic coupling of these advanced computational techniques specifically tailored for the structural and operational constraints of the IEEE 69-bus system remains underrepresented. This research addresses this gap by proposing a holistic architecture that synchronizes predictive intelligence with heuristic optimization to achieve superior grid performance^41^.

### Main contributions of the study

Although numerous studies have investigated either forecasting techniques or optimization strategies for Virtual Power Plants (VPPs), relatively few have developed a unified framework that integrates accurate data-driven forecasting with multi-objective optimization while considering the technical and operational constraints of practical distribution networks. Existing approaches often optimize a single performance aspect, such as economic operation or renewable energy scheduling, without simultaneously addressing network reliability, voltage regulation, operational costs, and environmental sustainability. Furthermore, comprehensive validation using realistic time-series operation, techno-economic assessment, and distribution-system performance metrics remains limited.

To address these research gaps, this paper proposes an integrated intelligent VPP management framework that combines machine learning, metaheuristic optimization, and comprehensive techno-economic analysis for coordinated operation of distributed energy resources (DERs). The proposed framework is designed to improve operational efficiency while ensuring scalability, reproducibility, and practical applicability in modern smart distribution networks.

The principal contributions and novelties of this work are summarized as follows:

- **Integrated Hybrid Forecasting–Optimization Framework**: A unified computational framework is developed by combining an Artificial Neural Network (ANN), trained using the Levenberg–Marquardt algorithm, with the Grey Wolf Optimizer (GWO). The ANN accurately forecasts hourly electricity demand, while the GWO simultaneously determines the optimal placement, sizing, and scheduling of Distributed Generation (DG) units and Battery Energy Storage Systems (BESS), enabling coordinated VPP operation under varying loading conditions.
- **Comprehensive Multi-Objective Energy Management**: Unlike conventional approaches that focus on a single objective, the proposed framework simultaneously optimizes multiple performance criteria, including active power loss minimization, voltage profile enhancement, renewable energy utilization, operating cost reduction, and CO₂ emission mitigation. This integrated formulation provides a balanced trade-off between technical reliability, economic profitability, and environmental sustainability.
- **Realistic Validation and Performance Evaluation**: The proposed methodology is validated using the IEEE 69-bus radial distribution system through both steady-state and time-series simulations. Comprehensive performance metrics are employed to evaluate network operation, including active power losses, voltage regulation, renewable energy utilization, energy storage scheduling, operational costs, discounted payback period (DPP), Net Present Value (NPV), Internal Rate of Return (IRR), and CO₂ emission reduction. Comparative analyses demonstrate the effectiveness and robustness of the proposed framework, achieving a **61.7% reduction in active power losses**, improving the minimum bus voltage from **0.909 p.u.** to **0.966 p.u.**, reducing daily operating costs by **$1**,**741.32**, and yielding a **3.8-year discounted payback period** with an **IRR of 30%**.

By integrating high-accuracy machine learning forecasting with metaheuristic multi-objective optimization and comprehensive techno-economic evaluation, the proposed VPP framework offers a robust, scalable, and practically deployable solution for intelligent energy management in future smart distribution systems with high renewable energy penetration.

The remainder of this paper is organized as follows. Section “Integrated VPP management framework” reviews the background and related literature while defining the research problem. Section “System mathematical formulation” presents the mathematical models of the system components, including photovoltaic (PV) generation, wind turbines (WTs), Battery Energy Storage Systems (BESS), load forecasting, and network constraints. Section “Multi-objective function formulation” formulates the multi-objective optimization problem and describes the implementation of the Grey Wolf Optimizer (GWO) within the proposed VPP energy management framework. Section “Grey wolf optimizer algorithm” presents the simulation setup, validation studies, comparative performance analysis, and techno-economic evaluation. Finally, Sect. 6 summarizes the main conclusions and outlines directions for future research.

## Integrated VPP management framework

The increasing penetration of Distributed Renewable Energy Sources (RES), particularly solar photovoltaic (PV) and wind turbines (WT), within modern distribution networks introduces substantial operational complexities. These challenges arise from the inherent intermittency, rapid variability, and stochastic uncertainty of such resources. These fluctuations propagate through the network, manifesting as increased dispatch errors, elevated imbalance costs, and compromised system reliability. In the absence of high-fidelity forecasting models, Virtual Power Plant (VPP) operators are unable to achieve optimal resource scheduling or efficient market participation^42^.

Given a radial distribution feeder characterized by candidate buses for Distributed Generation (DG) installation, variable load profiles, and uncertain renewable generation, this study aims to concurrently enhance three critical performance domains:

- Technical Performance: Minimization of active power losses and enhancement of voltage profiles.
- Economic Performance: Reduction of daily operational expenditures (OPEX) and improvement of financial metrics, including Internal Rate of Return (IRR), Net Present Value (NPV), and payback periods.
- Environmental Performance: Mitigation of CO_2_ emissions via maximized renewable utilization and reduced reliance on grid imports^43^.

The problem is decomposed into a Planning Stage, utilizing the Grey Wolf Optimizer (GWO) for optimal DG sizing, and an Operational Stage, employing a 24-hour time-series dispatch based on machine learning forecasts and scheduling. Validation is conducted via a forward-backward sweep power flow analysis^44^.

### Neural network (NN) forecasting module

The forecasting architecture utilizes a multi-layer perceptron (MLP) framework. The input data undergoes a normalization process to ensure computational stability as Eq. (1)^45^:

1 $$\:{X}_{N}=\:\frac{X-{\mu\:}_{X}}{{\sigma\:}_{X}}$$

Where * X* represents the raw input data (e.g., solar irradiance, wind speed, or electrical load), $\:{\mu\:}_{X}$ is the mean, and $\:{\sigma\:}_{X}\:$is the standard deviation. The transformation through the hidden layers is expressed as flow:

2 $$\:{\mathrm{h}}^{\mathrm{i}}=\sigma\:\:\left({W}^{\mathrm{i}}{X}_{N}+{b}^{\mathrm{i}}\right)$$

3 $$\:{\mathrm{h}}^{\mathrm{l}}=\sigma\:\:\left({W}^{\mathrm{l}\:}{\mathrm{h}}^{\mathrm{l}}+{b}^{\mathrm{l}}\right)$$

In these expressions, $\:{\mathrm{h}}^{\mathrm{l}}$ denotes the output of the * l-th* hidden layer for a total of * L* layers. $\:{W}^{\mathrm{l}\:}$and $\:{b}^{\mathrm{l}}$ represent the weight matrix and bias vector of the * l-th* layer, respectively, while $\:\sigma\:$ is the non-linear activation function. The final prediction * Y* is obtained by de-normalizing the raw network output$\:\:g$.

4 $$\:\mathrm{y}={\sigma\:}_{y}\mathrm{*}g+{\mu\:}_{y}$$

To minimize the discrepancy between predicted and actual values, the network is trained using the Mean Squared Error (MSE) loss function:

5 $$\:\mathrm{M}\mathrm{S}\mathrm{E}=\frac{1}{N}\sum\limits_{i=1}^{N}\:{\left({y}_{i}-{g}_{i}\right)}^{2}$$

For applications requiring probabilistic insights, a Quantile Loss function is implemented to estimate specific percentiles$\:\:{\uptau\:}$:

6 $$\:{J}_{\tau\:}\left(y,g\right)=\sum\limits_{i,y\ge\:{y}_{i}}^{.}{\uptau\:}\:\left|{y}_{i}-{g}_{i}\right|+\frac{1}{N}\sum\limits_{i,y\le\:y\_i}^{.}\:\left(1-\tau\:\right)\left|{y}_{i}-{g}_{i}\right|$$

Where * y* is the actual value, * g* is the predicted quantile, and $\:\tau\:\in\:\left[\mathrm{0,1}\right]$ specifies the desired quantile (e.g.,$\:\:\tau\:=0.5$ for the median).

### Evaluation metrics and uncertainty injection

To ensure robust VPP operation under stochastic conditions, forecasted values are augmented with confidence intervals derived from prediction errors. The available PV power at bus * i* and time * t* is constrained by an upper bound to account for uncertainty:

7 $$\:{PS}_{PV}\le\:{P}_{PV}+k*{\sigma\:}_{err}$$

Where $\:{PS}_{PV}$the scheduled power is output, $\:{P}_{PV}$ is the predicted output from the neural network, and $\:{\sigma\:}_{err}$ represents the standard deviation of the forecast error. This formulation allows the optimization engine to account for potential deviations, ensuring that the dispatch remains feasible even under realized load demand $\:{P}_{L}$fluctuations.

## System mathematical formulation

The proposed framework integrates the physical constraints of the distribution network with the operational dynamics of aggregated Distributed Energy Resources (DERs), including renewable generation and storage. The following assumptions are made in the mathematical formulation:

1. The distribution network is assumed to be balanced and operates under steady-state conditions.
2. The load is modeled as a constant power load model.
3. The PV and WT generation outputs are based on forecasted solar irradiance and wind speed data.
4. The BESS is assumed to have constant charging and discharging efficiencies.
5. The grid import/export prices are assumed time varying based on the electricity market.

### VPP power balance and network constraints

The fundamental requirement of the VPP is to maintain a continuous balance between power generation, storage, and demand. The instantaneous power balance for the VPP at time$\:\:t$ is defined as^46^:

8 $$\begin{aligned}\:{P}_{grid,t}+&\sum\:{P}_{PV,t}+\sum\:{P}_{WT,t}+\sum\:{P}_{DG,t}+{P}_{BESS,dis,t}\\&={P}_{load,t}+{P}_{grid,exp,t}+{P}_{BESS,ch,t}+{P}_{loss,t}\end{aligned}$$

where $\:{P}_{grid,t}$= Power imported from or exported to the grid at time $\:t{,P}_{PV,t},\:$PV power output at time * t*,$\:{P}_{WTt}$, Wind turbine power output at time * t*, Distributed Generation power output at time * t*,$\:\:{P}_{BESS,dis,t}$, BESS discharging power at time * t*, $\:{P}_{load,t}$, Load demand at time * t*,

. To ensure the technical integrity of the radial distribution system, the following power flow and operational limits are enforced using the Distribution Flow model^47^:

- **Active and Reactive Power Flow**:

9 $$\:{P}_{J,t}=\sum\limits_{k=J}{P}_{k,t}+{P}_{L,J,t}-{P}_{PV,J,t\:}-{P}_{WT,i,t}-{P}_{DG,J,t}-{P}_{BESS,J,t}+{R}_{i,j}\frac{{P}_{i,j,t}^{2}+{Q}_{i,j,t}^{2}}{{V}_{i,t}^{2}}$$

10 $$\:{Q}_{J,t}=\sum\limits_{k=J}{Q}_{k,t}+{Q}_{L,J,t}-{Q}_{DG,J,t}-{Q}_{BESS,q,J,t}+{X}_{i,j}\frac{{P}_{i,j,t}^{2}+{Q}_{i,j,t}^{2}}{{V}_{i,t}^{2}}$$

- **Voltage and Thermal Limits**:

11 $$\:{V}_{min}\le\:{V}_{i,t}\le\:{V}_{max}$$

12 $$\:{S}_{i,j,t}=\sqrt{{P}_{ij,t}^{2}+{Q}_{ij,t}^{2}}\le\:{S}_{i,j,max}$$

Where $\:{V}_{i}$ represents the voltage at bus* i*, and $\:{S}_{i,j,max}$ denotes the thermal capacity of the branch connecting buses * i* and * j*.

### DER device models and constraints

#### Photovoltaic (PV) systems

The active power output of PV units is a function of solar irradiance * G* and cell temperature$\:{T}_{cell}$^48^:

13 $$\:{P}_{PV,t}={P}_{STC}*{\eta\:}_{rel}*\frac{{G}_{t}}{1000}\left[1+\gamma\:\left({T}_{cell}-25\right)\right]){f}_{loss}$$

The inverter capacity restricts the combined active * P* and reactive * Q* power injection

14 $$\:\sqrt{{P}_{PV,t}^{2}+{Q}_{RES,t}^{2}}\le\:{S}_{inv,i}$$

#### Wind turbine (WT) systems

The power output of a wind turbine $\:{P}_{WT,t}$ is modeled as a piecewise function of the forecasted wind speed$\:{P}_{Wr}$: The produced power from the ($\:{P}_{WT}$) is determined as a function of the wind speed, its rated power $\:{P}_{Wr}$the rated wind speed ($\:{W}_{r}$), the cut-in wind speed $\:{W\:}_{i,}$ and the cut-out wind speed ($\:{W}_{o}$) from Eq^49^. :

15 $$\:{P}_{WTDG}=\left\{\begin{array}{ccc}0&\:\mathrm{\:for\:}&\:{W}_{\mathrm{wind\:}}<{W\:}_{i}\:\mathrm{o}\mathrm{r}\:{W}_{\mathrm{wind\:}}>{W}_{o}\\\:{P}_{Wr}\left(\frac{{W}_{\mathrm{wind\:}}-{W}_{i}}{{W}_{r}-{W}_{i}}\right)&\:\mathrm{\:for\:}&\:\left({W}_{i}\le\:{W}_{\mathrm{wind\:}}\le\:{W}_{r}\right)\\\:{P}_{wr}&\:\mathrm{\:for\:}&\:\left({W}_{r}<{W}_{\mathrm{wind\:}}\le\:{W}_{o}\right)\end{array}\right.$$

#### Battery Energy Storage System (BESS)

The State of Charge * SoC*dynamics and operational limits are governed by^50^:

16 $$\:SO{C}_{t}=SO{C}_{t-1}+\left({\eta\:}_{ch.}{P}_{ch,t}-\frac{{P}_{dis,t.}}{{\eta\:}_{dis}}\right){\Delta\:}t$$

17 $$\:SO{C}_{min}\le\:SO{C}_{t}\le\:SSO{C}_{max}$$

To preserve battery health, a complementarity constraint $\:{P}_{ch,t},{P}_{dis,t.\:}=0$ is enforced to prevent simultaneous charging and discharging.

## Multi-objective function formulation

The optimization engine seeks a Pareto-optimal solution across three weighted dimensions: technical, economic, and environmental^51^.

18 $$\:MinF={w}_{1}*{f}_{Tech}+{w}_{2}*{f}_{Econ}+{w}_{3}*{f}_{Env}$$

### Technical objective

$\:{f}_{Tech}$ Minimizes total system power losses $\:{P}_{loss}$and voltage deviation index* VDI*:

19 $$\:{f}_{Tech}=\sum\limits_{t=1}{P}_{loss,t}+\sum\limits_{t=1}^{24}\sum\limits_{i=1}^{N}{\left({V}_{i,t}-{V}_{ref}\right)}^{2}$$

### Economic objective

$\:{f}_{Econ}$ Minimizes total daily operating costs, including grid imports, DG fuel, and BESS degradation, while maximizing export revenue:

20 $$\:{f}_{Econ}=\sum\limits_{t=1}^{24}\left[\left({C}_{imp,t\:}{p}_{imp,t}-{C}_{exp,t}\:{P}_{exp,t}\right)+{C}_{DG}\:{P}_{DG,t}+{C}_{deg\:}\:\left|{P}_{BESS,t}\right|\right]$$

### Environmental objective

$\:{f}_{Env}$: Mitigates the carbon footprint based on emission factors$\:\in\:$ as Eq. (21).

21 $$\:{f}_{Env}=\sum\limits_{t=1}^{24}\left[{\in\:}_{grid}\:\left({p}_{imp,t}-{P}_{exp,t}\right)+{\in\:}_{DG}\:{P}_{DG,t}\right]$$

## Grey wolf optimizer algorithm

**Fig. 3:**
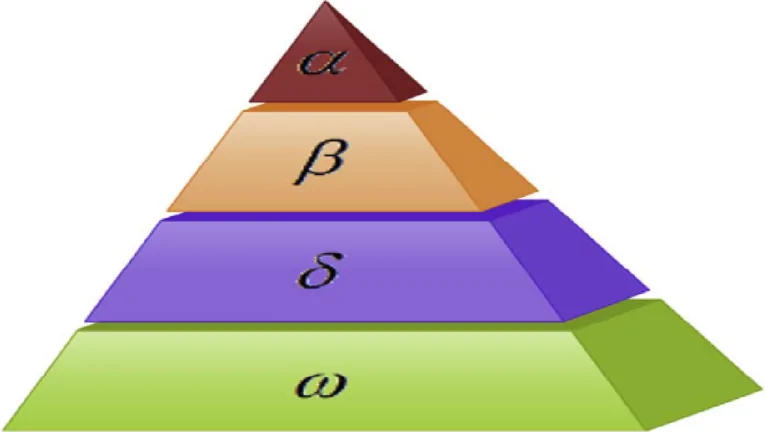
Hierarchy of grey wolves.

Grey wolves are predators that live in groups (packs). The pack of grey wolves has a special social hierarchy where the leadership in the pack is divided into four levels, which are alpha, beta, omega, and delta. Alpha wolf (α) is the first level in the social hierarchy; hence, alpha wolf is the leader who guides the pack, and the other wolves respond to its orders. Beta wolf (β) is in the second level of leadership, where it helps the alpha wolf with the activities of the pack. Delta (δ) wolves come in the third level of hierarchy, following α and β wolves^25^. The rest of the wolves are the omegas (ω), which submit to the whole group Fig. 3^17^.

### Social hierarchy

The best solution is considered as the alpha (α). Hence, the second and the third solution are considered as β and δ, and the other solutions will be treated as ω wolf.

### Surrounding the prey

The grey wolves encircle the prey in the hunting process, which can be mathematically modeled as follows:

22 $$\:\mathrm{D}=\left|\mathrm{C}.{\mathrm{k}}_{\mathrm{p}}\left(\mathrm{t}\right)-\mathrm{X}\left(\mathrm{t}\right)\right|$$

23 $$\:\mathrm{X}\left(\mathrm{t}+1\right)={\mathrm{X}}_{\mathrm{p}}\left(\mathrm{t}\right)-\mathrm{A}.\mathrm{D}$$

Where$\:\:\mathrm{t}$ is the current iteration, $\:{\mathrm{X}}_{\mathrm{p}}$ is the position vector of the prey, and$\:\:\mathrm{X}$ indicates the position vector of a grey wolf.$\:\:\mathrm{A}$ and$\:\:\mathrm{C}$ are coefficient vector which can be calculated as follows by the Core update equations^52^

24 $$\:\mathrm{A}=2\mathrm{a}.{\mathrm{r}}_{1}-\mathrm{a}$$

25 $$\:\mathrm{C}=2.{\mathrm{r}}_{2}$$

where$\:\:\mathrm{a}$ is a value that is decreased linearly from 2 to 0 with iterations.$\:{\mathrm{r}}_{1}$and$\:{\:\mathrm{r}}_{2}$are random numbers in the range [0, 1].

### Hunting the prey

In the hunting process, the pack is affected by$\:\:{\upalpha\:}$, $\:{\upbeta\:}$ and δ; hence, the first three best solutions are saved as best agents ($\:{\upalpha\:}$,$\:{\upbeta\:}$, δ), and the other search agents update their positions according to the best agents as follows:

26 $$\:\mathrm{D}=\left|\mathrm{C}.{\mathrm{X}}_{\mathrm{p}}\left(\mathrm{t}\right)-\mathrm{X}\left(\mathrm{t}\right)\right|$$

27 $$\:{\mathrm{D}}_{{\upalpha\:}}=\left|{\mathrm{C}}_{1}.{\mathrm{X}}_{{\upalpha\:}}-\mathrm{X}\right|$$

28 $$\:{\mathrm{D}}_{{\upbeta\:}}=\left|{\mathrm{C}}_{2}.{\mathrm{X}}_{{\upbeta\:}}-\mathrm{X}\right|$$

29 $$\:{\mathrm{D}}_{{\updelta\:}}=\left|{\mathrm{C}}_{3}.{\mathrm{X}}_{{\updelta\:}}-\mathrm{X}\right|$$

30 $$\:{\mathrm{X}}_{1}={\mathrm{X}}_{{\upalpha\:}}-{\mathrm{A}}_{1}.\left({\mathrm{D}}_{{\upalpha\:}}\right)$$

31 $$\:{\mathrm{X}}_{2}={\mathrm{X}}_{{\upbeta\:}}-{\mathrm{A}}_{2}.\left({\mathrm{D}}_{{\upbeta\:}}\right)$$

32 $$\:{\mathrm{X}}_{3}={\mathrm{X}}_{{\updelta\:}}-{\mathrm{A}}_{3}.\left({\mathrm{D}}_{{\updelta\:}}\right)$$

33 $$\:\mathrm{X}\mathrm{n}\mathrm{e}\mathrm{w}=\frac{{\mathrm{X}}_{1}+{\mathrm{X}}_{2}+{\mathrm{X}}_{3}}{3}$$

### Attacking the prey

The last stage in hunting is attacking the prey, where the grey wolf attacks when the prey stops moving. It can be achieved mathematically by reducing the value of $\:\mathrm{a}$ gradually from 2 to 0; consequently, $\:\mathrm{A}$ is varied randomly with variation of $\:\mathrm{a}$, and it will be in the range [−1, 1], hence the next location of search agents will be between its current position and the position of the prey.

## Results and discussion

This section presents a comprehensive evaluation of the proposed Integrated Virtual Power Plant (VPP) framework, utilizing the IEEE 69-bus distribution system over a 24-hour operational horizon.

### Test system and computational environment

**Fig. 4:**
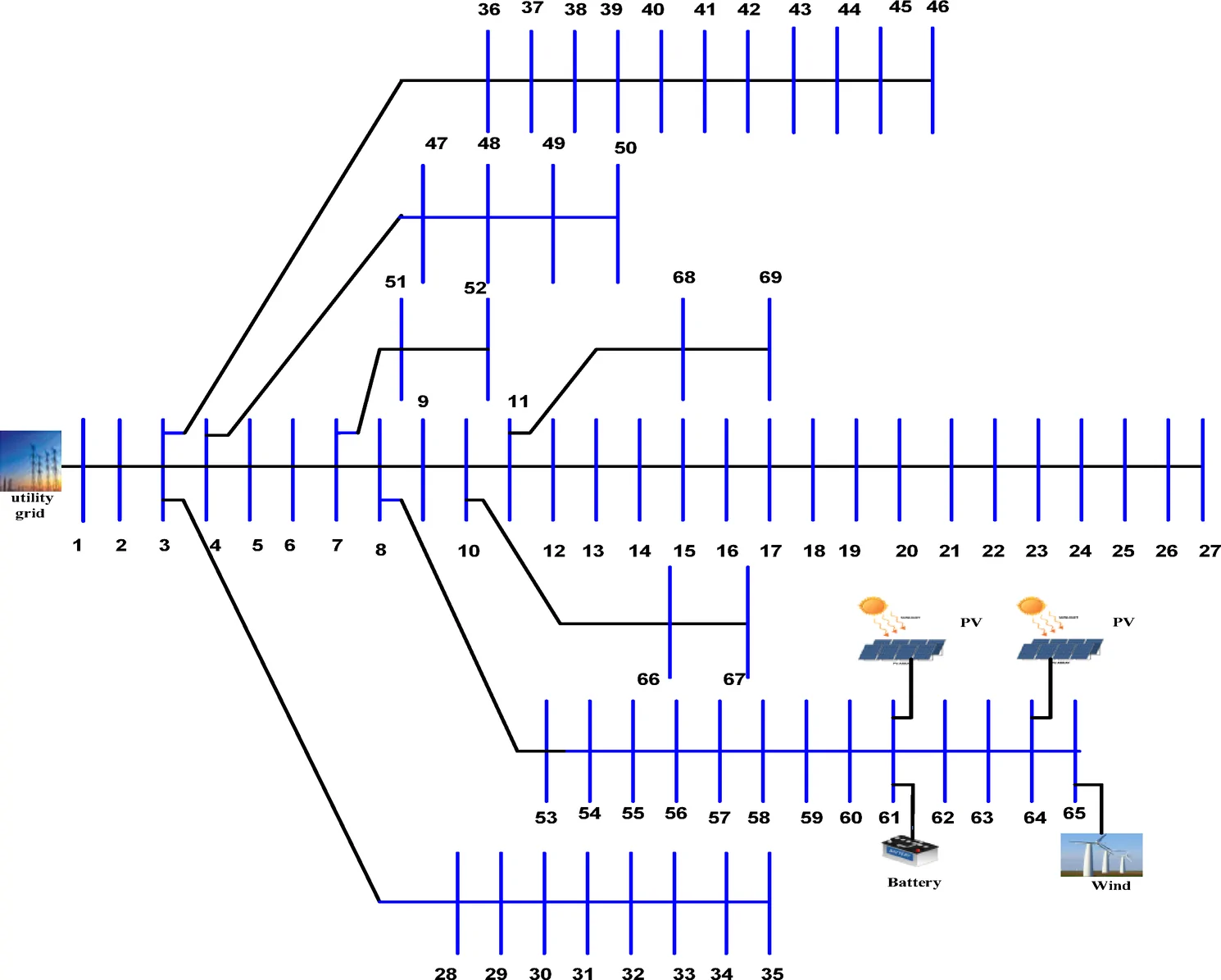
The single-line diagram of the 69-bus RDS.

The integrated NN-GWO framework was validated on the IEEE 69-bus radial distribution test system, a benchmark widely utilized in Distributed Generation (DG) planning. This system provides a realistic representation of real-world distribution networks characterized by high $R/X$ ratios and significant sensitivity to DG placement. The network configuration comprises 69 buses and 68 branches, with a total connected active load of 3.80 MW and a reactive load of 2.69 MVAr. The slack bus is positioned at bus 1 with a nominal voltage of 12.66 kV. In accordance with standard distribution system modeling, a static power load model is assumed^53^. The architectural layout of the system is illustrated in Fig. 4. All simulations were implemented in MATLAB 2018b and executed on a workstation equipped with an Intel^®^ Core™ i7 processor (3.20 GHz) and 32.0 GB of RAM. Reproducibility Details: The following parameters were used in the implementation:

- **BFS Power Flow**: convergence tolerance = 1e-6, maximum iterations = 100.
- **Forecasting**: training data = 80% of dataset, validation = 10%, test = 10%.

The dataset and code used for this study are available upon request from the corresponding author.

### Forecasting performance and energy profile

The ensemble neural network forecast effectively captures the diurnal demand pattern, demonstrating appropriate smoothing at peak extremes, which aligns with established SVM and ANN performance benchmarks^54^. The training trajectories exhibit early convergence and generalization saturation, confirming the model’s efficacy for day-ahead scheduling^55^. Over the simulation horizon, the energy yield from renewable resources is recorded as follows:

- Solar PV: 2.9 MWh (exhibiting a standard daytime bell-shaped output).
- Wind Turbine (WT): 1.3 MWh (contributing primarily during shoulder hours).

The total renewable energy availability of 4.2 MWh demonstrates the complementary nature of PV and WT resources, which enhances the VPP’s operational flexibility. The proposed hybrid approach achieves superior accuracy; with a mean prediction error of 1.27 MW compared to 1.60 MW for SVM and 2.10 MW for LSTM, representing a 20.6% improvement over SVM and a 39.5% improvement over LSTM show in Table 1.

**Table 1:** Forecasting performance comparison.

Model	Mean Prediction Error (MW)	RMSE (MW)	MAE (MW)
Proposed NN-GWO	1.27	0.15	0.12
SVM	1.60	0.21	0.18
Random Forest	1.80	0.24	0.20
LSTM	2.10	0.28	0.23

### Power flow and optimization parameters

The steady-state energy flow calculations were resolved using the Backward/Forward Sweep (BFS) algorithm^38,39^, selected for its robust convergence and proven affinity for radial distribution topologies^56,57^, The technical specifications, including line parameters and baseline power flow results (calculated in the absence of Renewable Energy Sources (RES)), are summarized in Tables 2, 3 and 4. Furthermore, the parameters selected for the optimization and machine-learning algorithms are detailed in the subsequent sections, ensuring the reproducibility of the study.

**Table 2:** System specification and initial power flow.

Item	Value	Value
System Specifications:		
$\:\mathbf{N}\mathbf{B}$	33	69
$\:\mathbf{N}\mathbf{p}\mathbf{r}$	32	68
Vsys (kV)	12.66	12.66
Base MVA	100	100
$\:{\mathbf{S}}_{\mathbf{L}\mathbf{o}\mathbf{a}\mathbf{d}}\left(\mathbf{M}\mathbf{V}\mathbf{A}\right)$	3.715 + j2.300	3.802 + j2.694
$\:{\mathbf{P}}_{\mathbf{T}\mathbf{o}\mathbf{t}\mathbf{a}{\mathbf{l}}_{\mathbf{l}\mathbf{o}\mathbf{s}\mathbf{s}}}\left(\mathbf{k}\mathbf{W}\right)$	210.84	225
$\:{\mathbf{Q}}_{\mathbf{T}\mathbf{o}\mathbf{t}\mathbf{a}{\mathbf{l}}_{\mathbf{l}\mathbf{o}\mathbf{s}\mathbf{s}}}\left(\mathbf{k}\mathbf{V}\mathbf{a}\mathbf{r}\right)$	143.022	102.198
$\:{\mathbf{V}}_{\mathbf{m}\mathbf{i}\mathbf{n}}\:(\mathbf{p}.\mathbf{u})$ @ bus	0.90378, 18	0.90919, 65

### Algorithmic and machine learning

The performance of the proposed framework is contingent upon the precise calibration of the optimization and forecasting parameters. The following tables delineate the configurations for the Grey Wolf Optimizer (GWO) and the comparative Machine Learning (ML) models. The neural network architecture utilized in the proposed framework was benchmarked against several state-of-the-art machine learning and deep learning models to ensure predictive accuracy^58^.

**Table 3:** Configuration parameters for the optimization framework.

Reference	Parameter Settings	Algorithm
^52^	T_max_=100, Search agents No. = 30, aa decreases linearly from 2 to 0, r1,r2∼U(0,1)r1,r2∼U(0,1)	GWO
Proposed	Tmax = 100, Search agents No. = 30, aa decreases linearly from 2 to 0, r1,r2∼U(0,1)r1,r2 U(0,1), Neural network: 3 layers [64,128,64], Levenberg-Marquardt training, 6 epochs	NN-GWO (Proposed)

**Table 4:** Parameter specifications for forecasting models.

Reference	Parameter Settings	Algorithm/Model
^23^	Hidden layers = 3, Neurons = [64, 128, 64], Activation = ReLU, Training algorithm = Levenberg-Marquardt, Epochs = 6, Loss function = MSE, Normalization = (x-µ)/σ	Neural Network (NN)
^59^	Ensemble method = Random Forest, Number of trees = 100, Max depth = 10, Min samples split = 5	Random Forest
^60,61^	SVM kernel = RBF, C = 1.0, Gamma = ‘scale’, Epsilon = 0.1	Support Vector Machine (SVM)
^62^	LSTM layers = 2, Units = [100, 50], Dropout = 0.2, Optimizer = Adam, Epochs = 50, Batch size = 32	LSTM (Deep Learning)

The integration of distributed generation (DG) units resulted in a notable improvement in the voltage profile across the distribution network, with all buses maintaining voltage magnitudes above 0.95 p.u and following DG placement. This outcome verifies the suitability and effectiveness of the selected DG locations. A forward–backward sweep load-flow analysis was conducted for both the base system and the DG-integrated configuration. In the base case (without DG), the total real power losses were 0.0023 MW, with a minimum bus voltage of 0.9092 p.u. Conversely, when optimized DG units were incorporated, total real power losses decreased to 0.0009 MW, and the minimum bus voltage increased to 0.9664 p.u., corresponding to a loss reduction of 61.74%. Moreover, two machine-learning models, Support Vector Machine (SVM) and Artificial Neural Network (ANN), were developed using meteorological and temporal input features. Their performance was subsequently evaluated using the designated test dataset^63^.

### Convergence and voltage profile

**Fig. 5:**
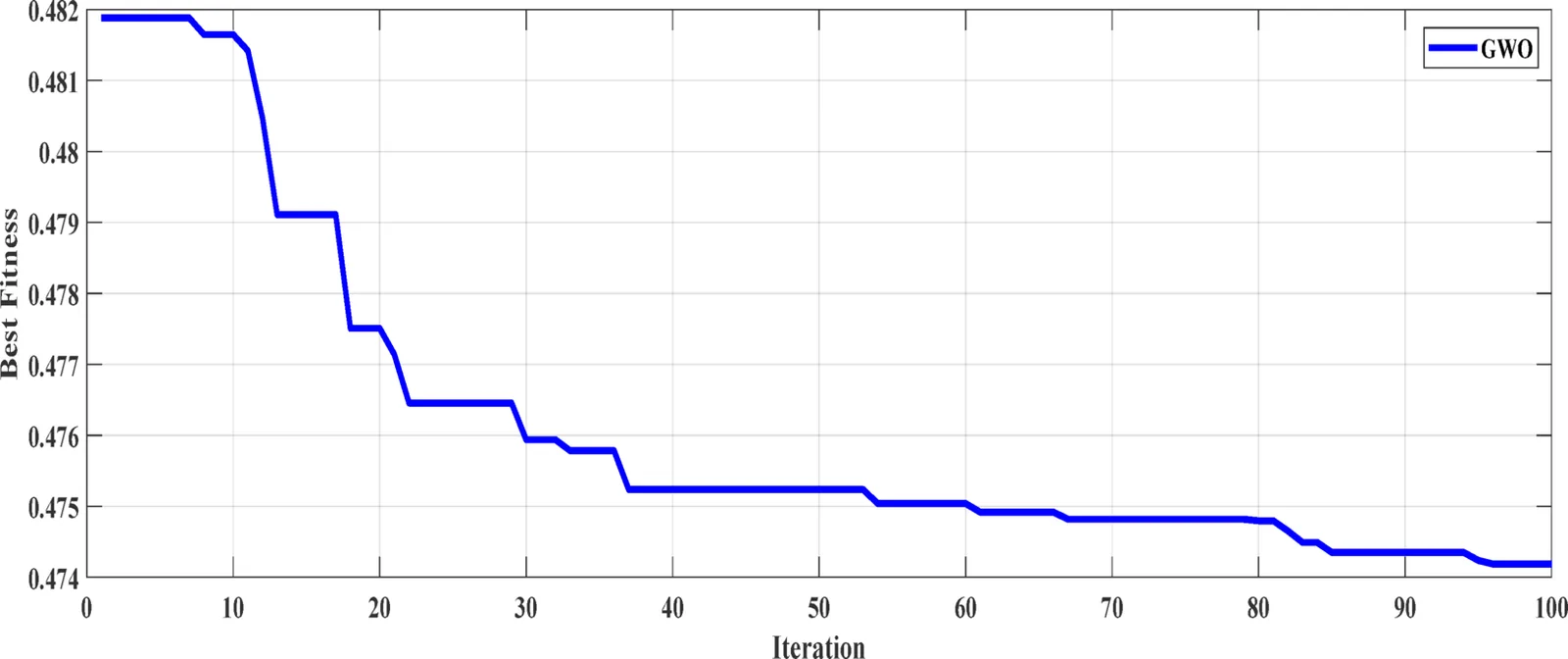
Convergence curve.

**Fig. 6:**
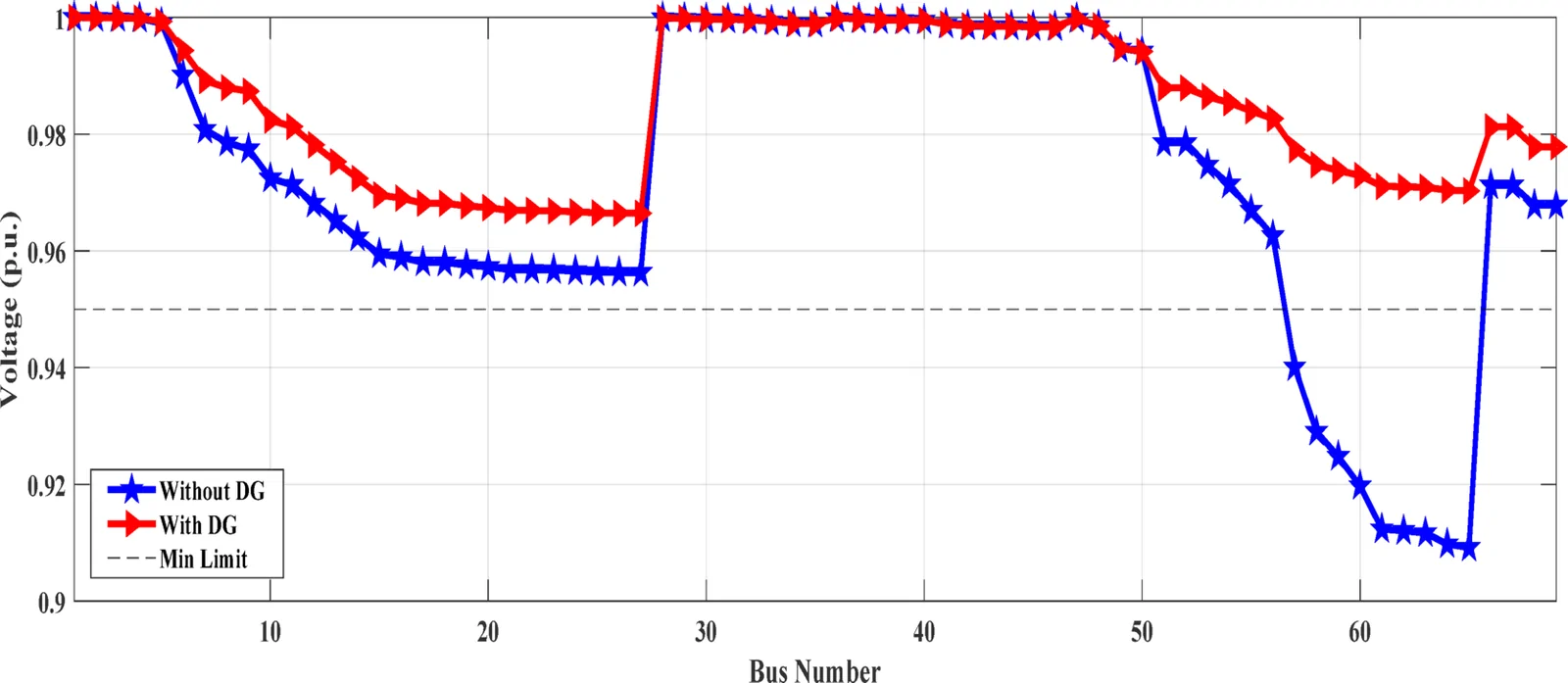
Voltage profile with and without DG.

Figure 5 depicts the convergence characteristics of the Grey Wolf Optimizer (GWO) during DG sizing. The best-fitness value decreases smoothly from approximately 0.482 to 0.4742 over 100 iterations, indicating stable, non-oscillatory convergence and supporting the algorithm’s reliability in attaining a high-quality optimum. Figure 6 compares the bus voltage profile before and after integrating the optimized DG units. In the base case, pronounced voltage drops are observed—particularly at downstream buses (0.91 p.u.). Following DG installation, the minimum voltage increases to 0.966 p.u., and the overall profile exhibits improved smoothness.

### ML-enhanced load forecasting

#### Forecast behavior and accuracy

**Fig. 7:**
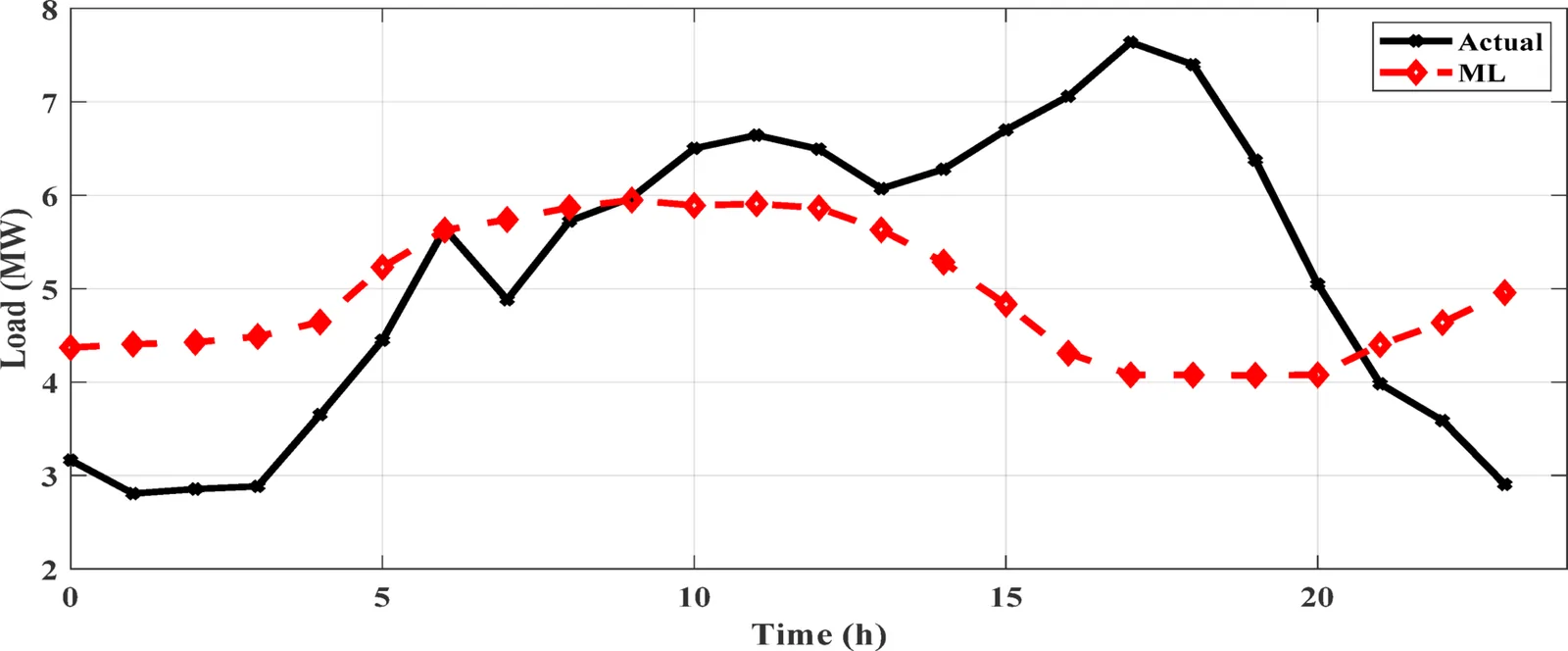
ML‑predicted load.

The observed improvement in voltage regulation and feeder loading is consistent with the recorded loss reduction of approximately 61.7%. Figure 7 presents the actual load trajectory alongside the machine-learning (ML) forecast. The ML prediction captures the general demand pattern but exhibits smoothing and underestimation near peak hours, consistent with the test-set error magnitudes (e.g., Support Vector Machine, SVM, and 1.6 MW). Despite deviations at extremes, the forecast accuracy is adequate for operational planning within the virtual power plant (VPP) framework.

### PV/WT physical generation

#### Wind and solar resource profiles

**Fig. 8:**
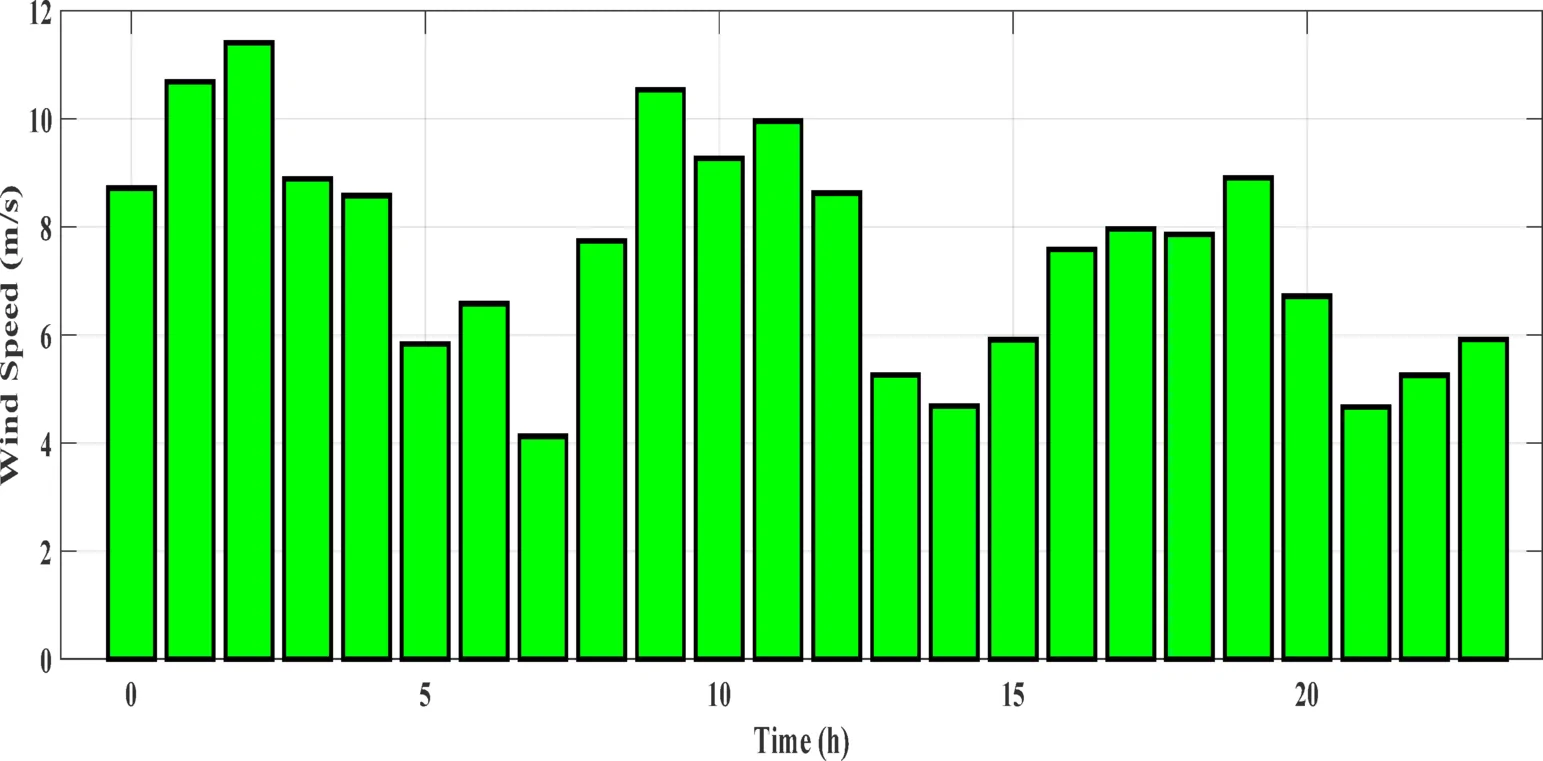
Wind speed profile.

**Fig. 9:**
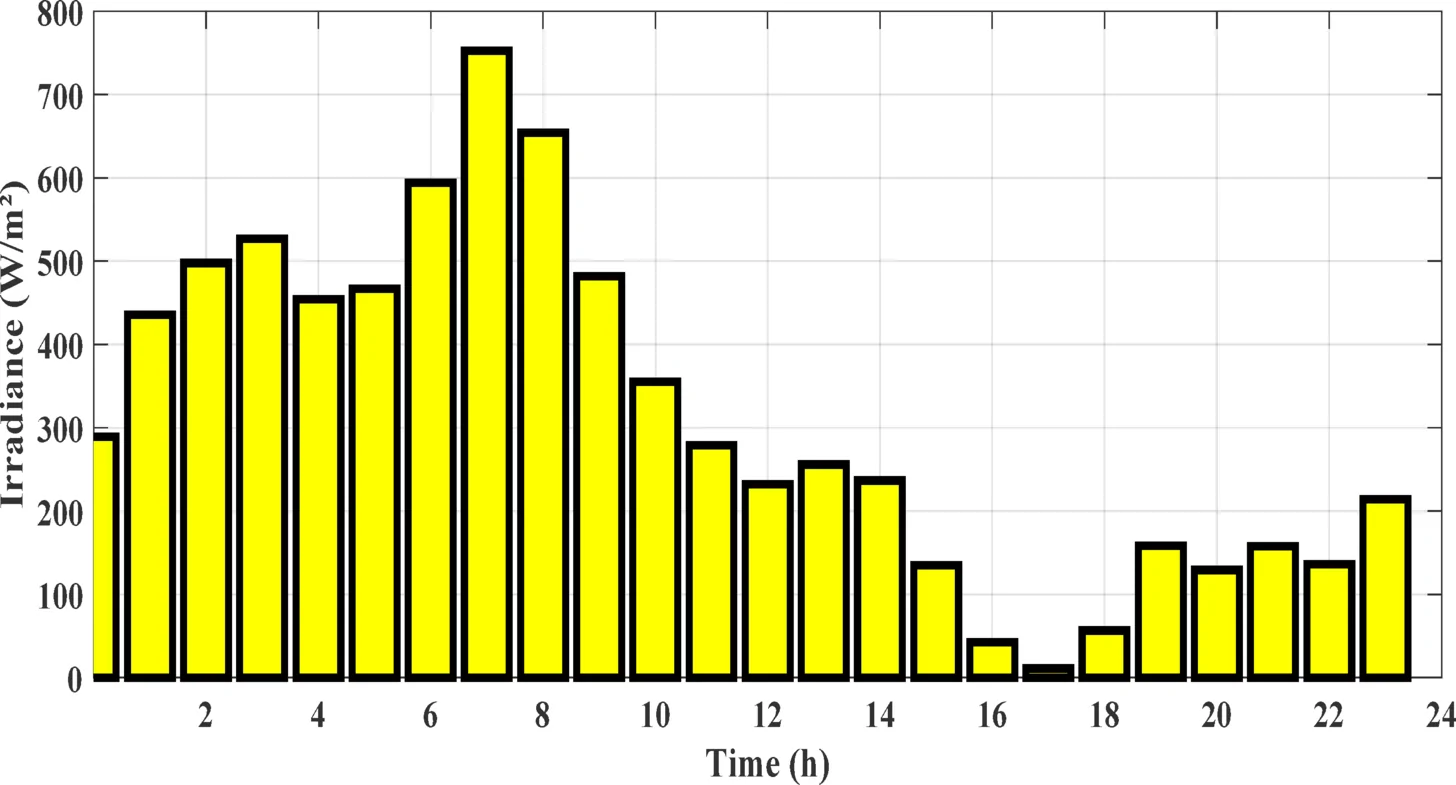
Solar irradiance profile.

Figure 8 shows wind speed varying between 4 and 11 m/s, with multiple periods exceeding the turbine cut-in and rated thresholds, thereby enabling sustained wind energy contribution. The observed variability underscores the intermittent nature of wind resources and substantiates the incorporation of a to ensure stable power availability. Figure 9 illustrates diurnal solar irradiance, increasing from early morning to a peak exceeding 750 W/m² around hours 8–9, then declining toward evening. This smooth irradiance pattern directly governs PV output and corroborates the daytime generation behavior assumed in the PV model.

### Optimal sizing of distributed generation

#### DG placement and capacities

The GWO was employed to determine the optimal capacities of distributed generation units at five candidate buses. The optimizer converged stably within 100 iterations, achieving a minimum objective value of 0.4742. The solution favors allocation near far-end, high-loss locations, particularly bus 61 show in Table 5.

**Table 5:** Optimal DG capacities.

Bus	DG Size (MW)
61	1.2442
62	0.0412
63	0.0000
64	0.2276
65	0.0621

The proposed method outperforms existing approaches, achieving the highest loss reduction (61.7%) and the best voltage profile (0.966 p.u.) show in Table 6.

**Table 6:** Comparative analysis of DG placement performance.

Study	Loss Reduction		Minimum Voltage (*p*.u.)		Voltage Improvement
Proposed Method	61.7%	0.966		+ 0.057	
Sultana et al.^24,25^	54.3%	0.948		+ 0.039	
Bouchikhi et al.^29–32^	58.1%	0.955		+ 0.046	
Alyu et al.^28^	59.5%	0.958		+ 0.049	

#### Renewable generation and forecast error

**Fig. 10:**
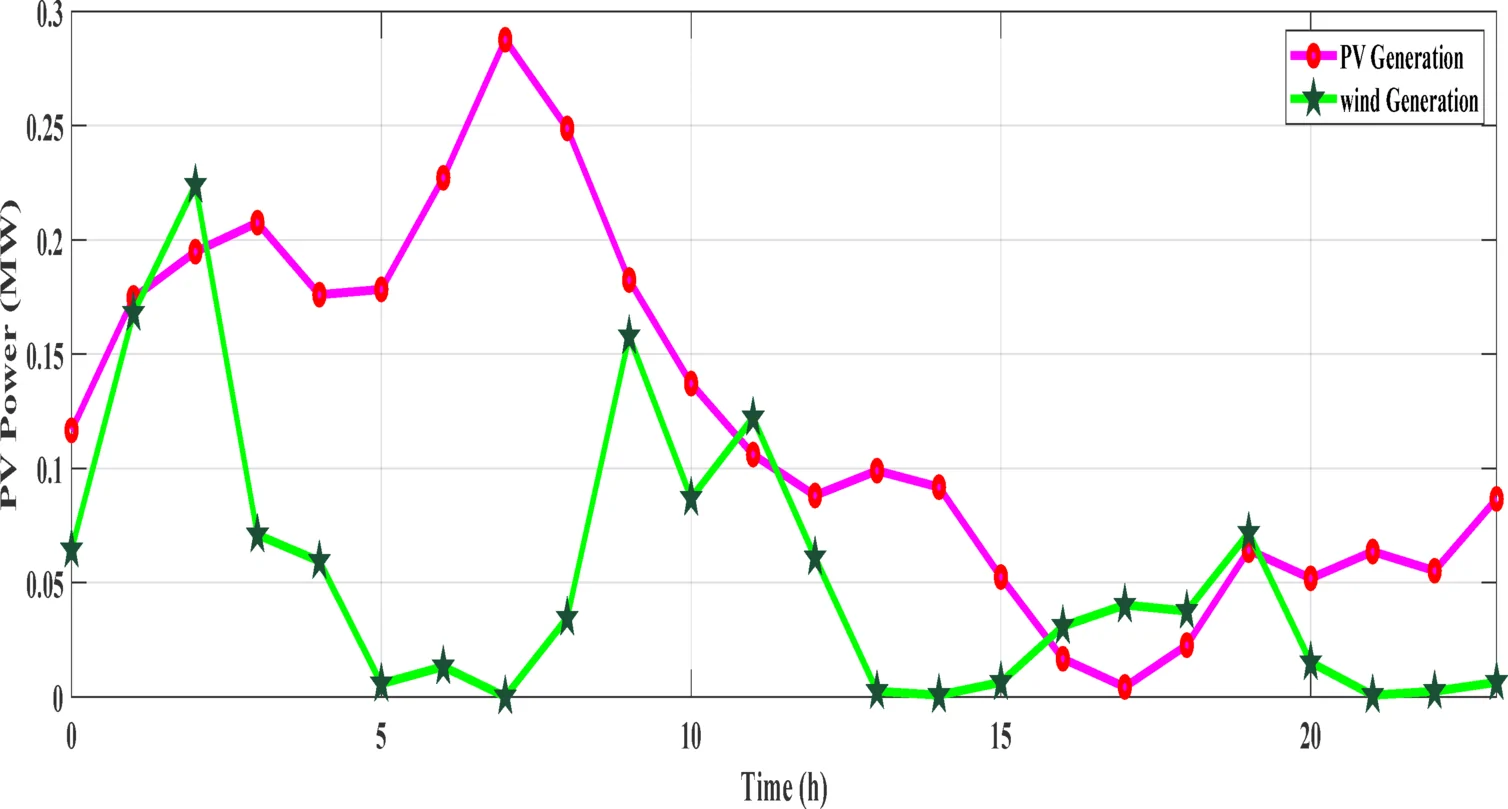
PV and Wind generation profiles.

**Fig. 11:**
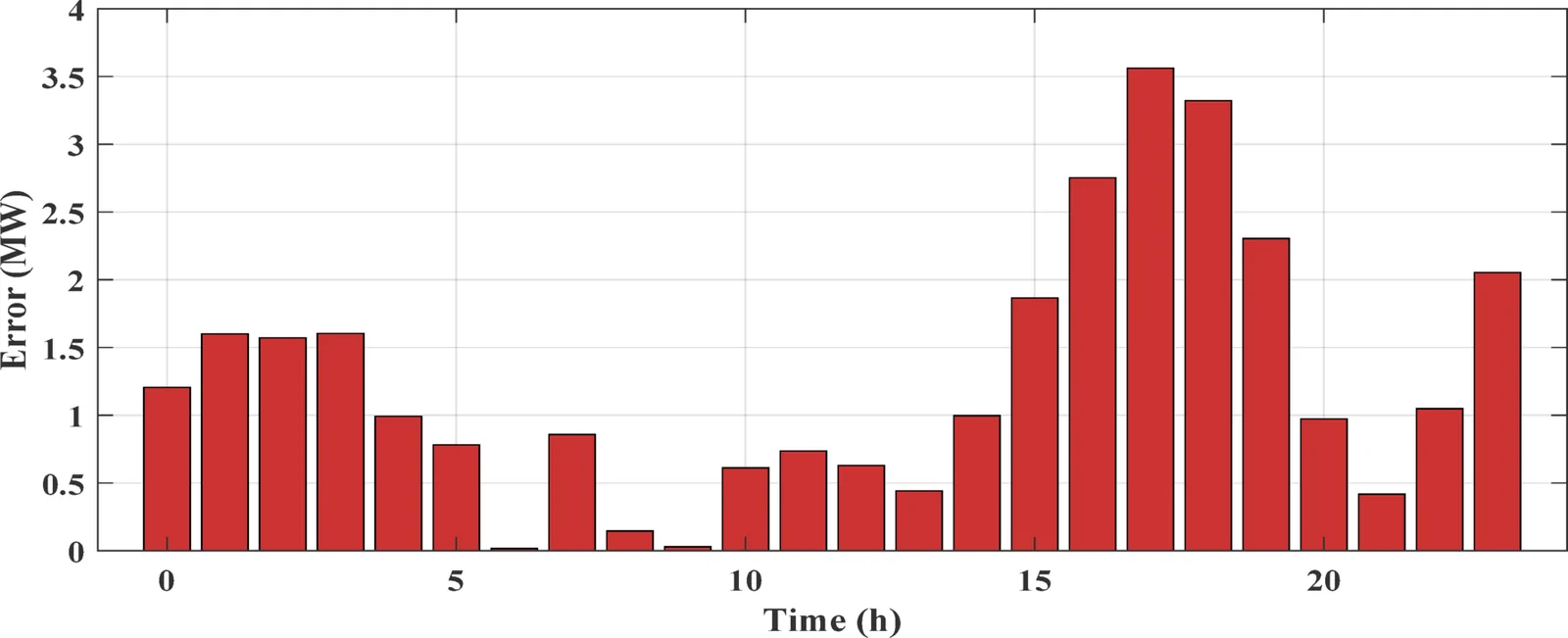
Forecasting error.

Figure 10 depicts the hourly power outputs of PV and wind. PV production follows a typical diurnal profile, increasing from sunrise to a midday peak of 0.29 MW and then tapering off toward sunset. Wind generation is more erratic due to natural intermittency, with short-term peaks between 0.05 and 0.22 MW. The complementary behavior of wind and PV is evident: wind contributes during early morning and late evening when PV output is minimal, thereby enhancing the VPP’s aggregate renewable availability. Figure 11 reports the absolute forecasting error between the ML-predicted and actual loads. Errors are relatively small during early morning hours but increase during peak-load periods (hours 15–19), exceeding 3 MW at times, which indicates reduced model accuracy during rapid demand transitions. Nonetheless, performance remains acceptable for operational planning.

#### Battery scheduling and state of charge

**Fig. 12:**
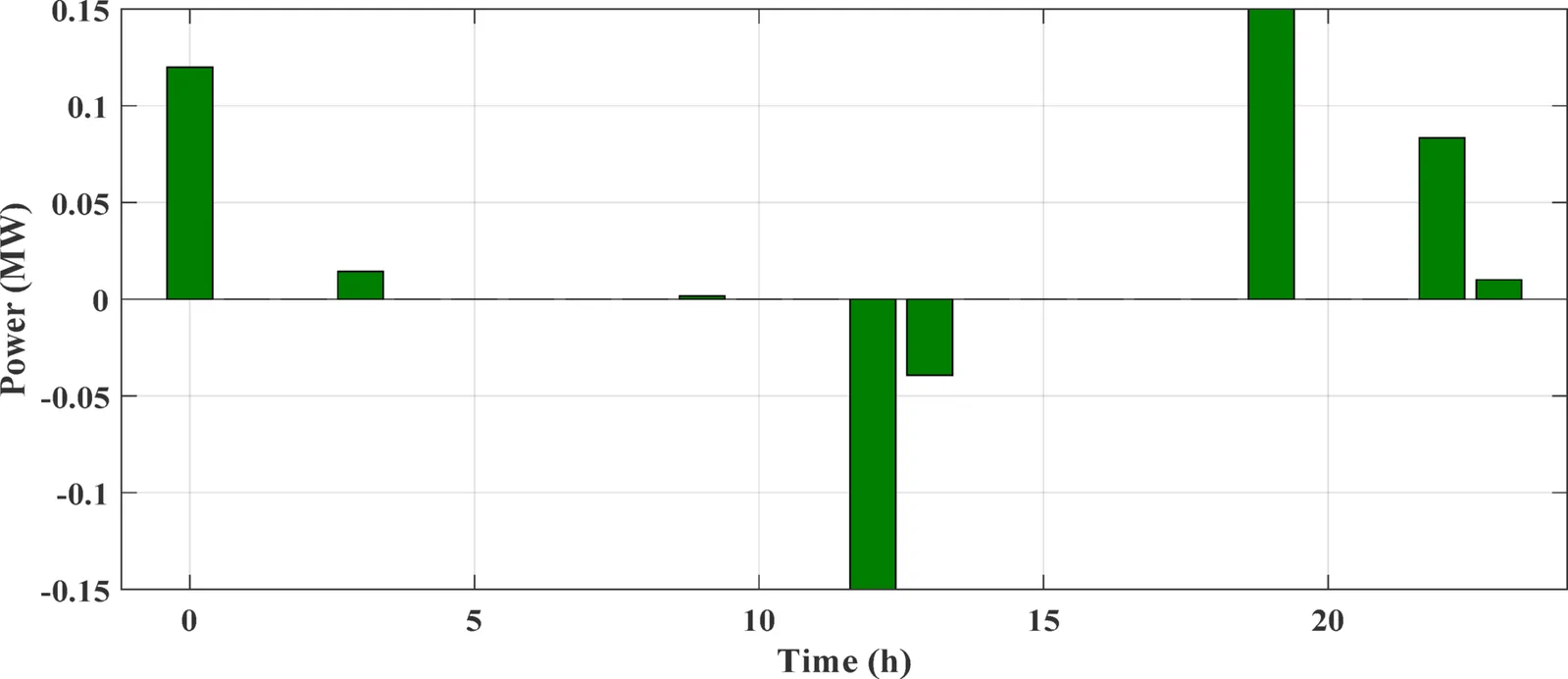
Battery charging and discharging.

**Fig. 13:**
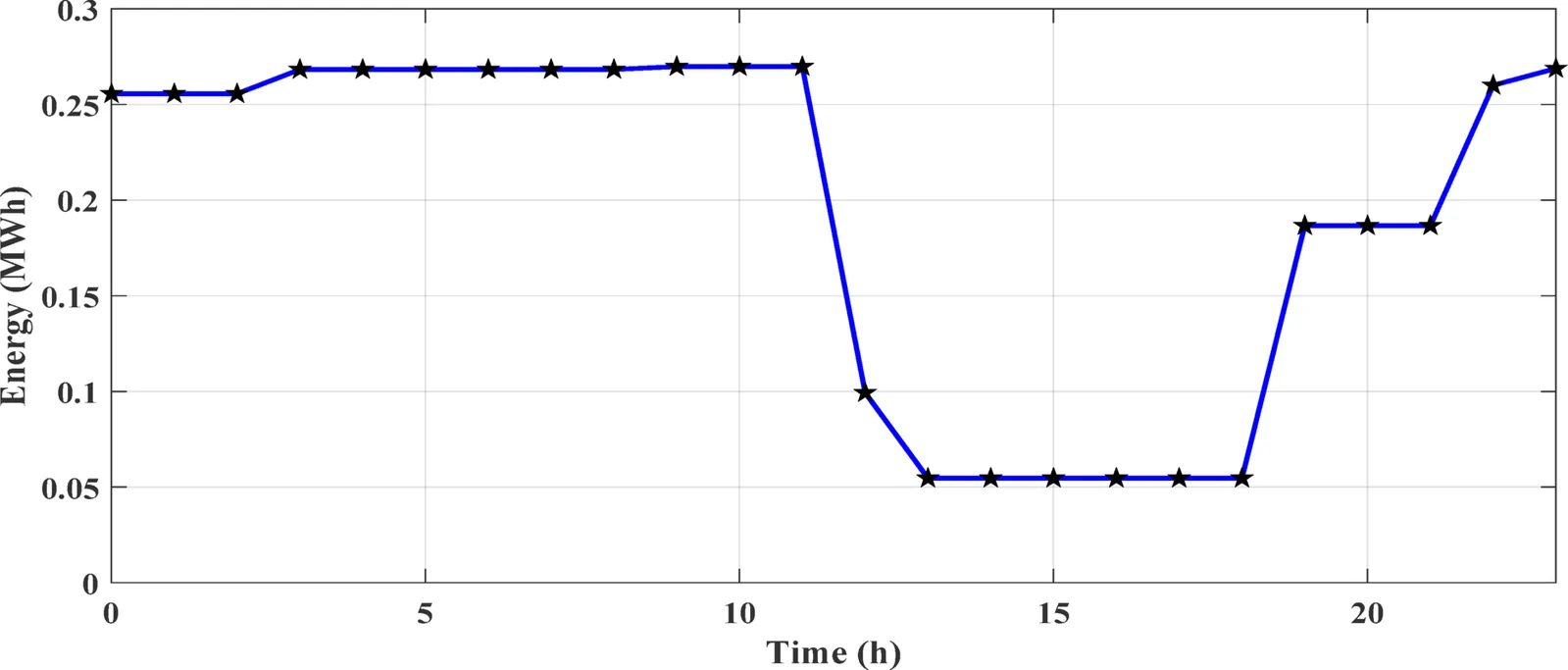
State of charge.

Consistent with price-based arbitrage logic, the BESS charges during low-price/low-demand intervals (early morning and late evening) and discharges during high-demand or high-price windows (e.g., hours 12–14), as illustrated in Fig. 12. Figure 13 shows that the state of charge (SOC) starts near 0.25 MWh, increases during charging periods, and then decreases markedly around midday coincident with high load. The BESS operates within its allowable limits, demonstrating effective energy shifting aligned with system needs.

## ANN training dynamics

**Fig. 14:**
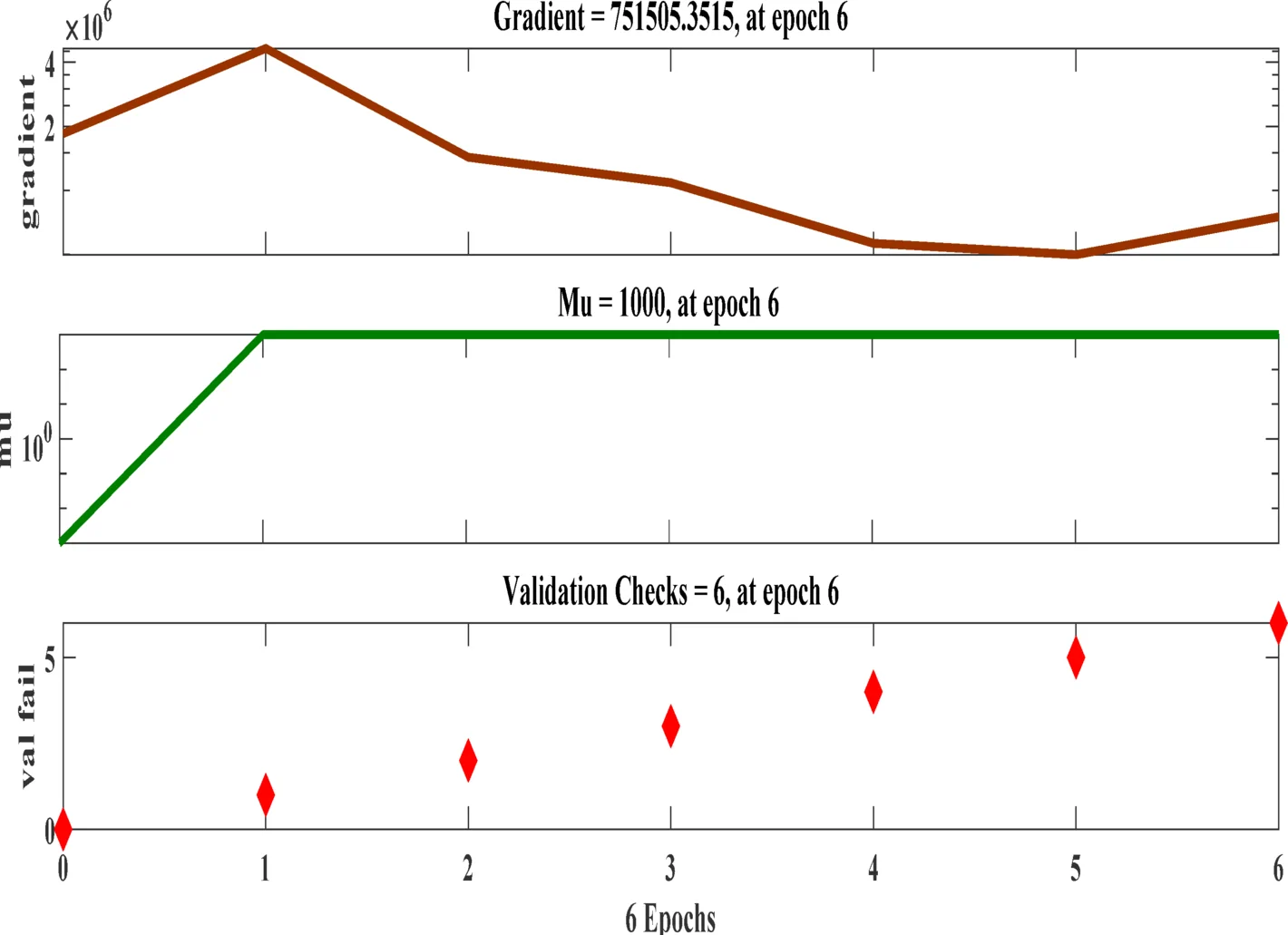
ANN training indicators.

**Fig. 15:**
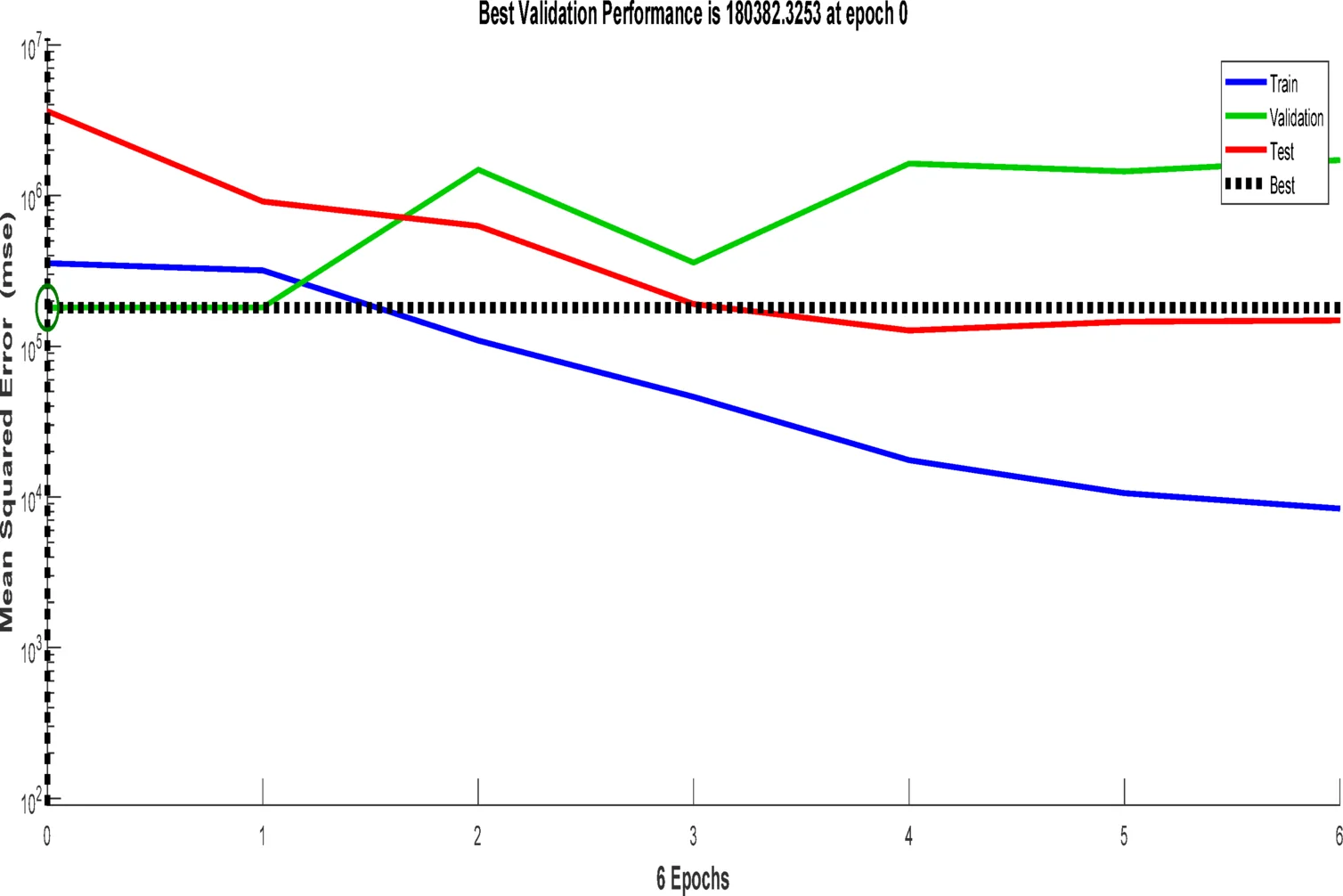
Mean squared error (MSE) for training-validation-testing.

**Fig. 16:**
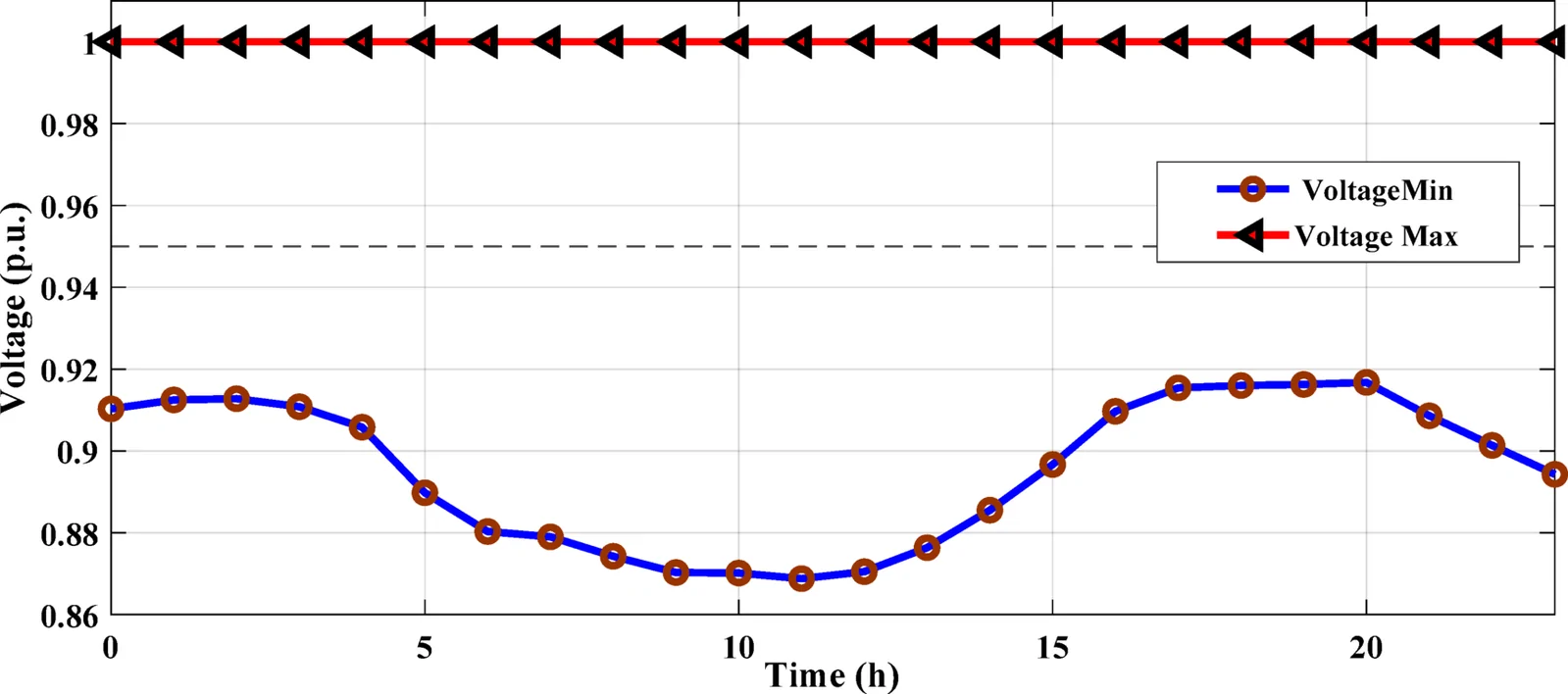
Minimum and maximum voltage.

During artificial neural network (ANN) training, the gradient initially increases and then gradually decreases, indicating progressive stabilization of weight updates. The damping parameter µ rises to its maximum, suggesting that the Levenberg–Marquardt routine entered a damped-update regime to prevent divergence. Validation checks trigger the stopping criterion at epoch 6, implying no further generalization gains beyond this point; overall, the model converges rapidly but exhibits early saturation (Fig. 14). Figure 15 presents the mean squared error (MSE) for the training, validation, and test sets. While the training error decreases monotonically, the validation and test errors fluctuate, with the best validation performance occurring at epoch 0, which indicates early overfitting i.e., the model fits the training data more readily than unseen samples. Even so, the absolute error levels remain acceptable for forecasting applications. Under dynamic loading, the minimum voltage dips to 0.868 p.u. during peak periods, confirming localized stress at downstream buses. Figure 16 shows the hourly minimum and maximum network voltages; the maximum remains near 1.0 p.u. throughout the day, indicating stable upstream conditions. These observations highlight the value of coordinated DG and BESS support to preserve voltage compliance under time-varying demand.

### Power losses and grid exchange

#### Network losses

**Fig. 17:**
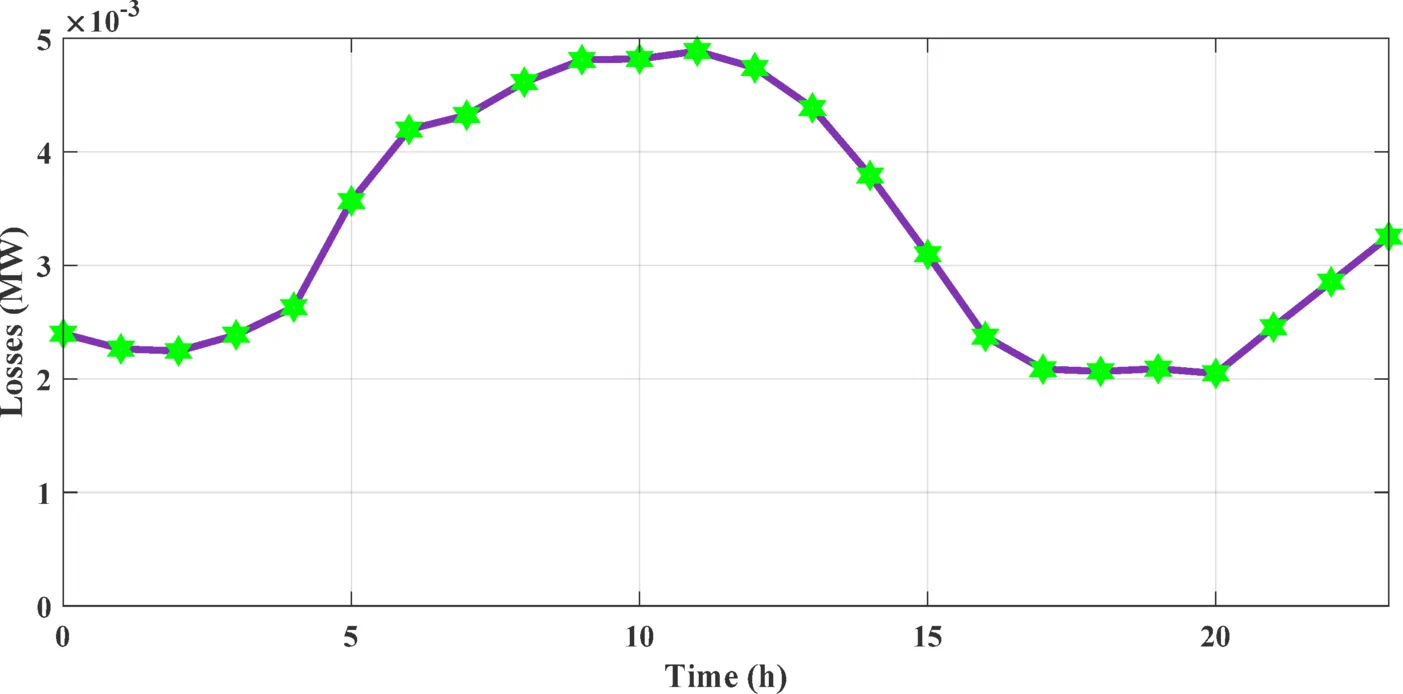
Power losses.

As shown in Fig. 17, active power losses increase around midday, approaching 5 × 10⁻³ MW, coincident with peak demand and comparatively reduced renewable contribution, and then decline during evening and early-morning low-load periods.

#### Grid interchange

**Fig. 18:**
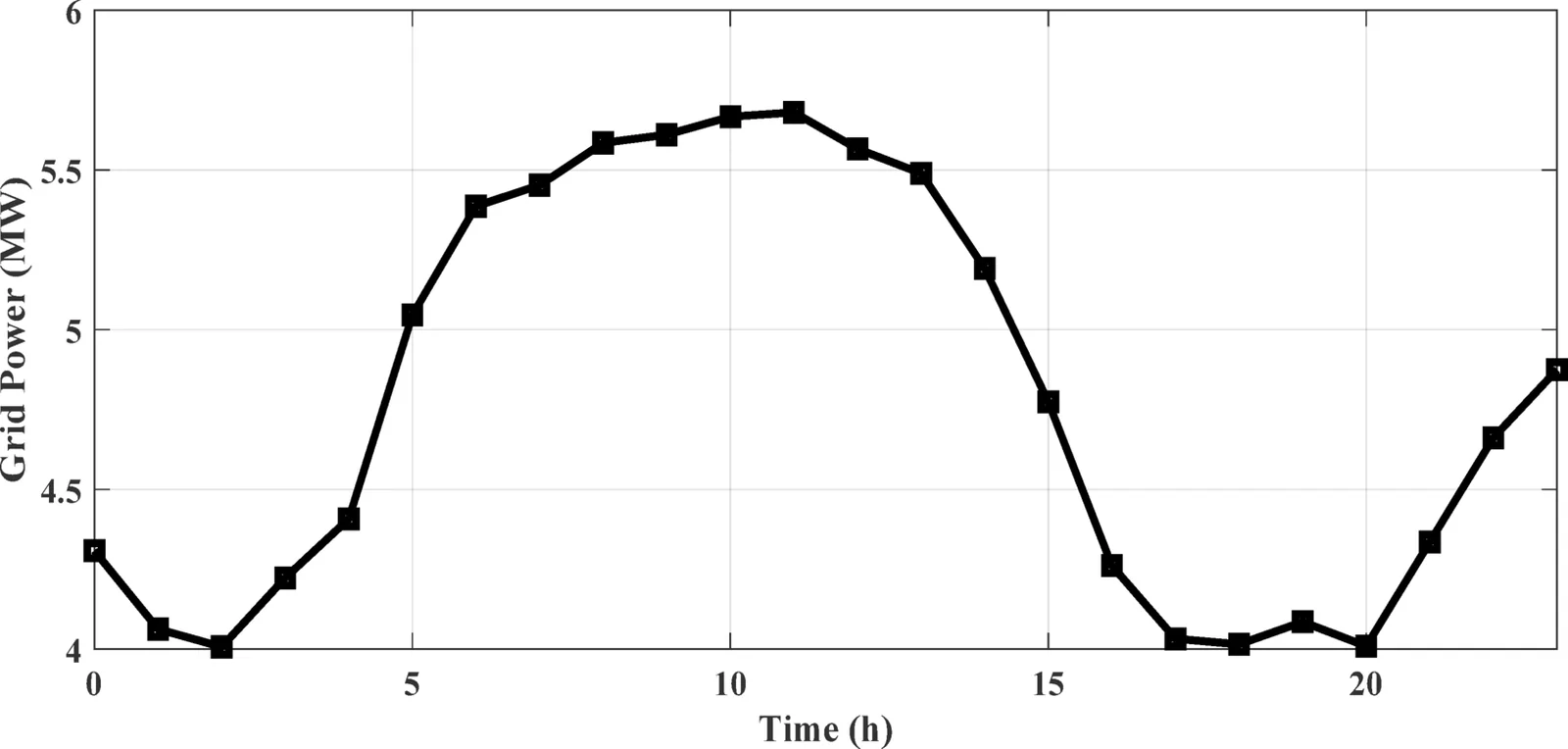
Grid power exchange.

Figure 18 displays the net power exchange between the VPP and the utility grid. Imports rise during the morning and peak around hours 10–12 (5.7 MW), reflecting greater reliance on the grid during high-load intervals. Exchange decreases sharply in the late evening due to lower demand coupled with contributions from renewables and the BESS. This behavior demonstrates that the VPP modulates grid dependency over the daily cycle.

## Techno-economic and environmental assessment

### Operational cost and investment value

**Fig. 19:**
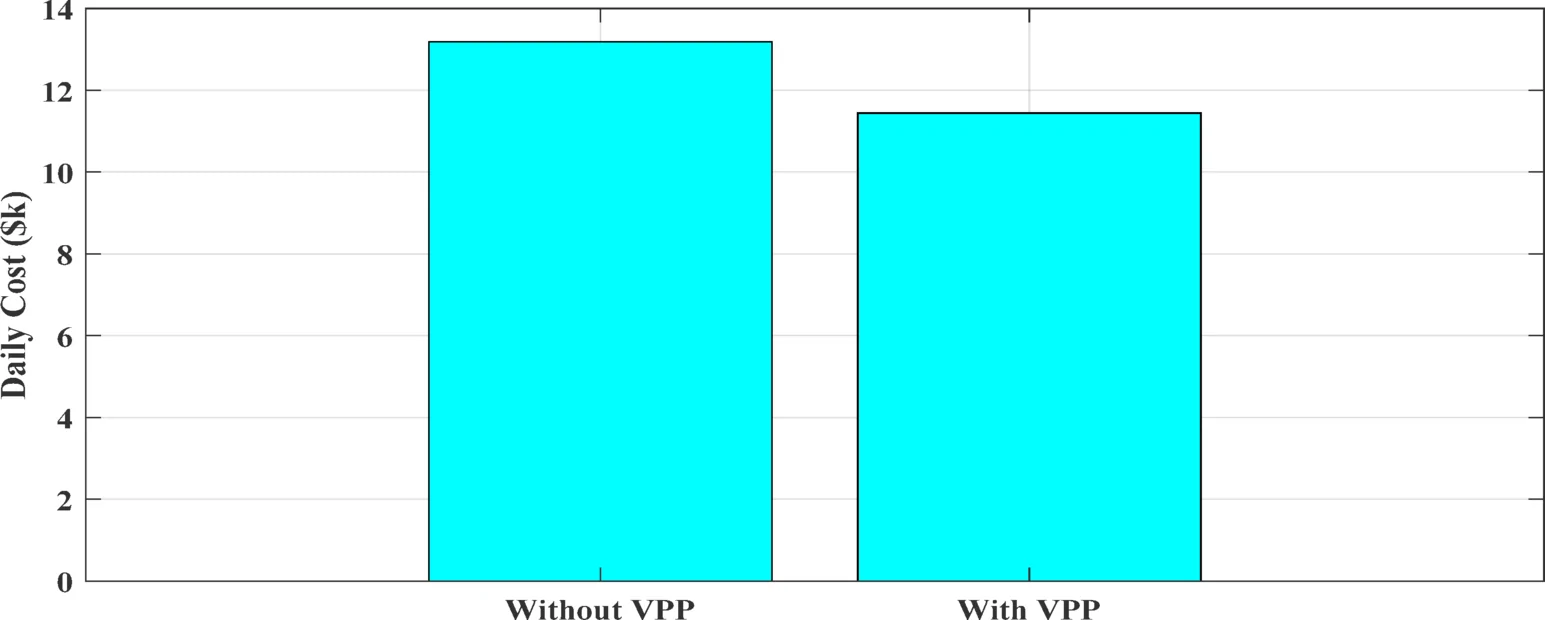
Cost with and without VPP.

**Fig. 20:**
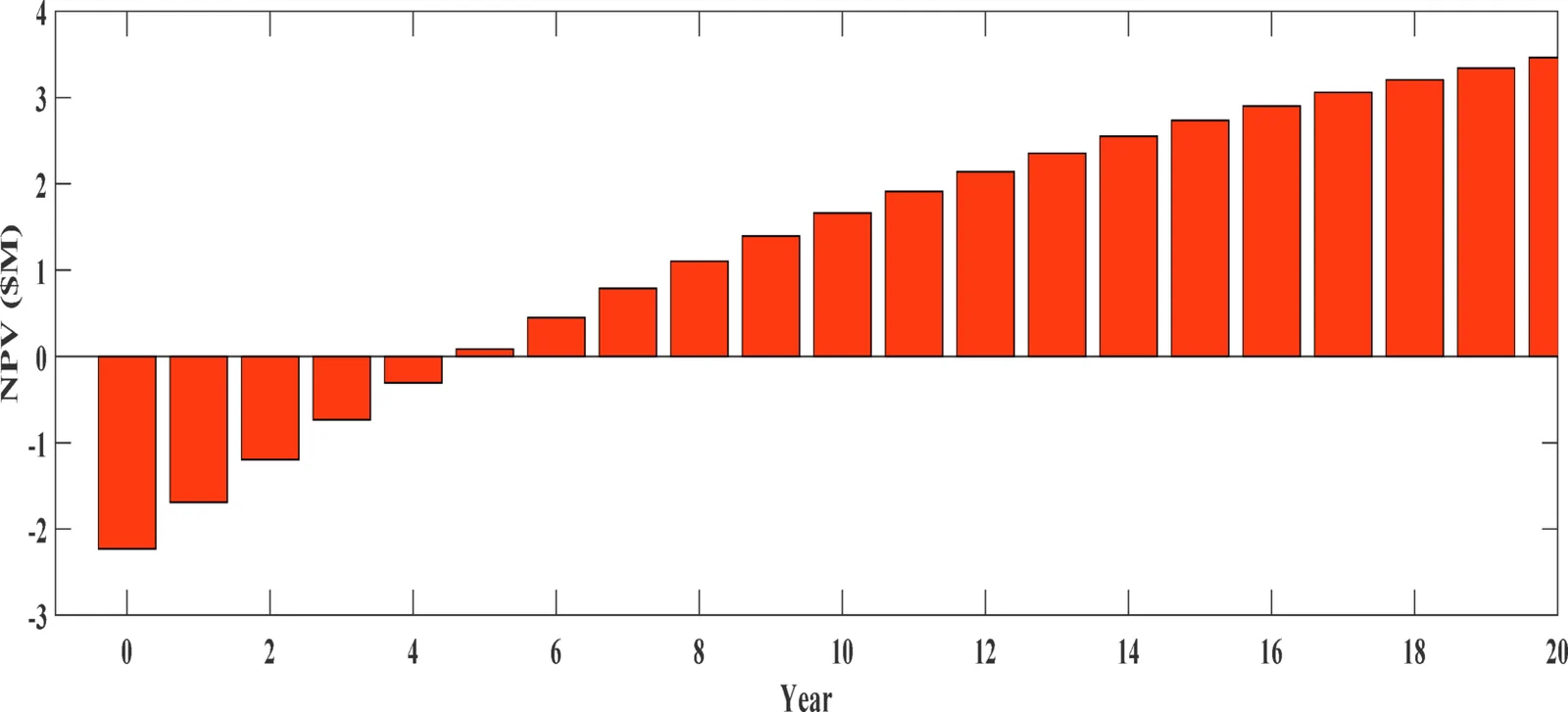
Net present value.

Figure 19 compares the total operating cost under conventional operation versus VPP-enabled coordination. The VPP scenario reduces daily expenditure from approximately $13k to about $11.5k, confirming that coordinated scheduling of distributed energy resources (DERs) decreases grid imports while leveraging local renewables. The net present value (NPV) trajectory in Fig. 20 indicates long-term economic viability: after initial negative values reflecting capital outlays, the curve crosses breakeven near year 5 and exceeds $3 million by year 20, signifying strong annual cash flows and an attractive investment outlook.

### Price signal and BESS strategy

**Fig. 21:**
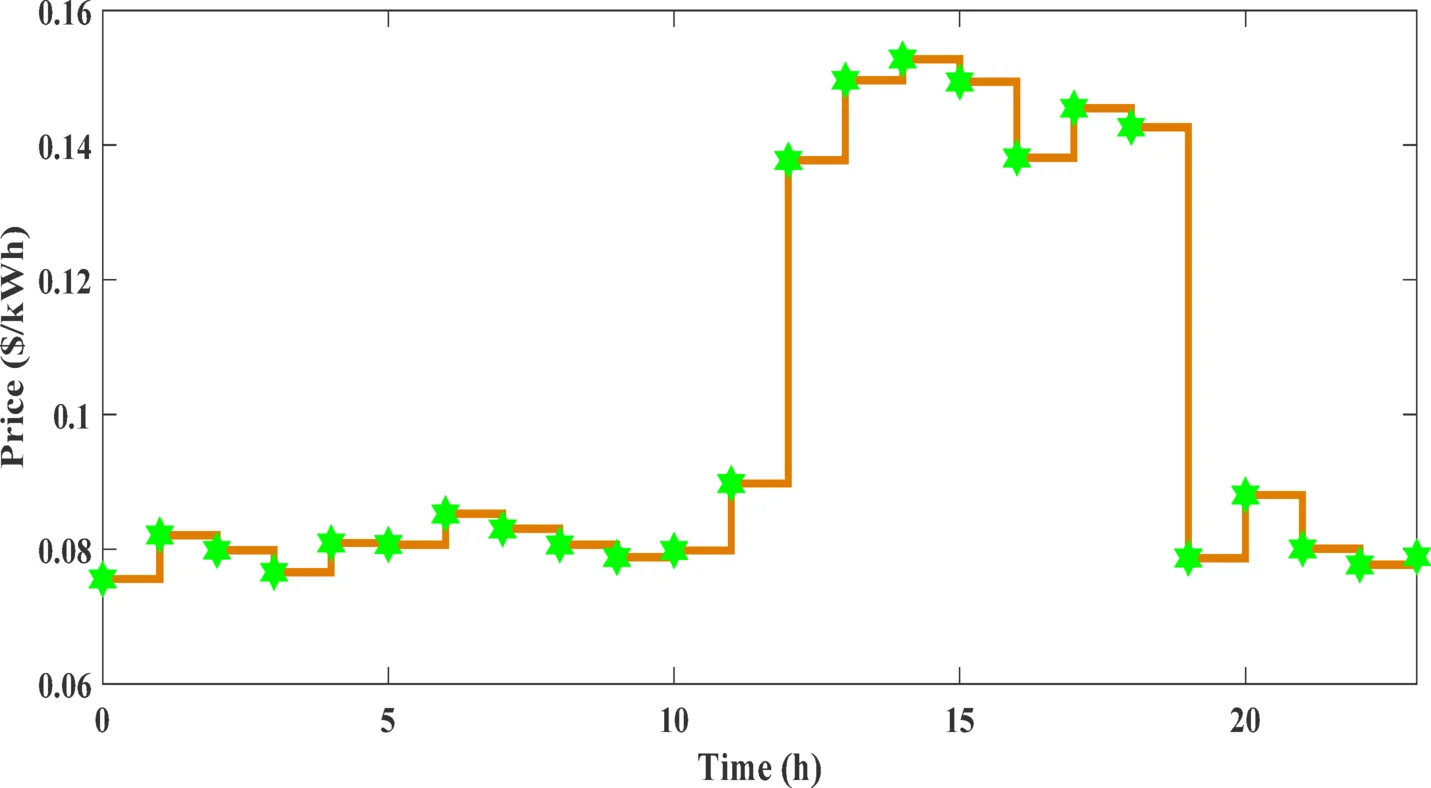
Electricity price.

As shown in Fig. 21, the hourly energy price features two distinct tariff zones: off-peak (low price) and a peak window between hours 12 and 18 (high price). This structure incentivizes strategic BESS operation charging during off-peak hours and discharging during peak tariffs thereby improving economic returns and mitigating grid stress.

### Emissions

**Fig. 22:**
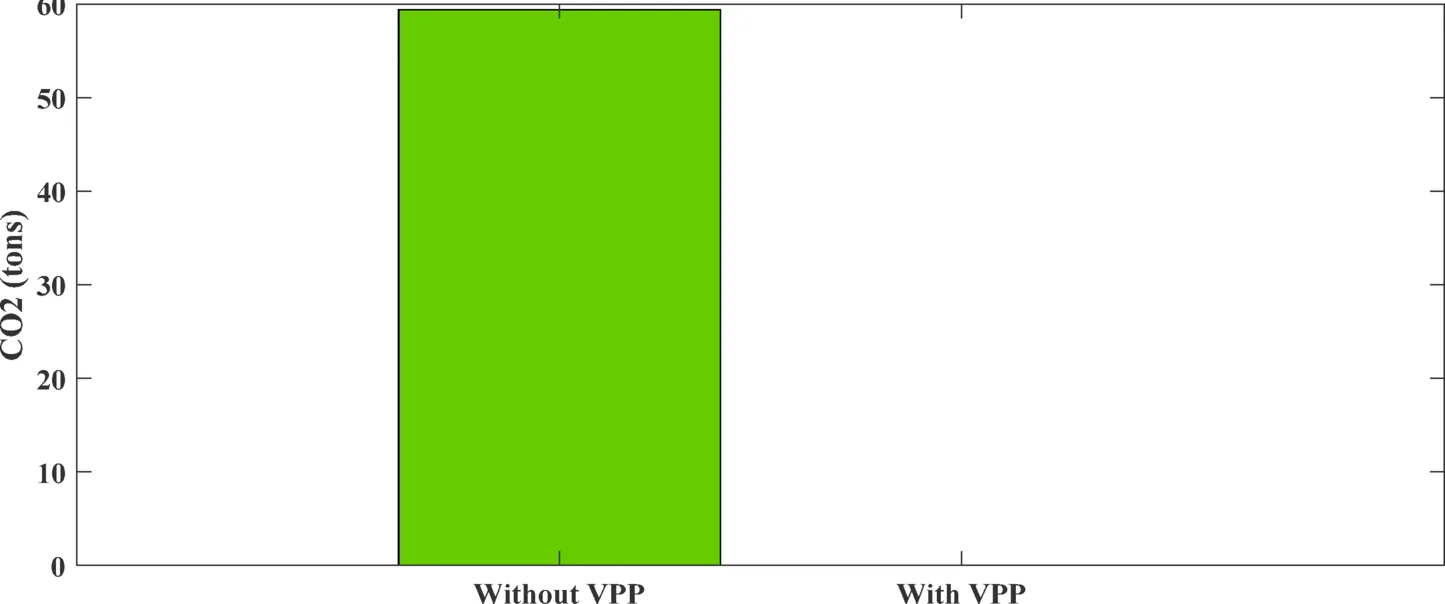
Emissions with and without VPP.

Figure 22 contrasts daily CO₂ emissions under the baseline (grid-dependent) case versus the VPP-enhanced framework. The baseline emits nearly 60 tons of CO₂ per day, whereas the VPP case effectively eliminates emissions due to high renewable penetration and a reduced reliance on fossil-fuel-based grid energy, underscoring the environmental advantages of VPP integration.

.

## Technical and battery performance

**Fig. 23:**
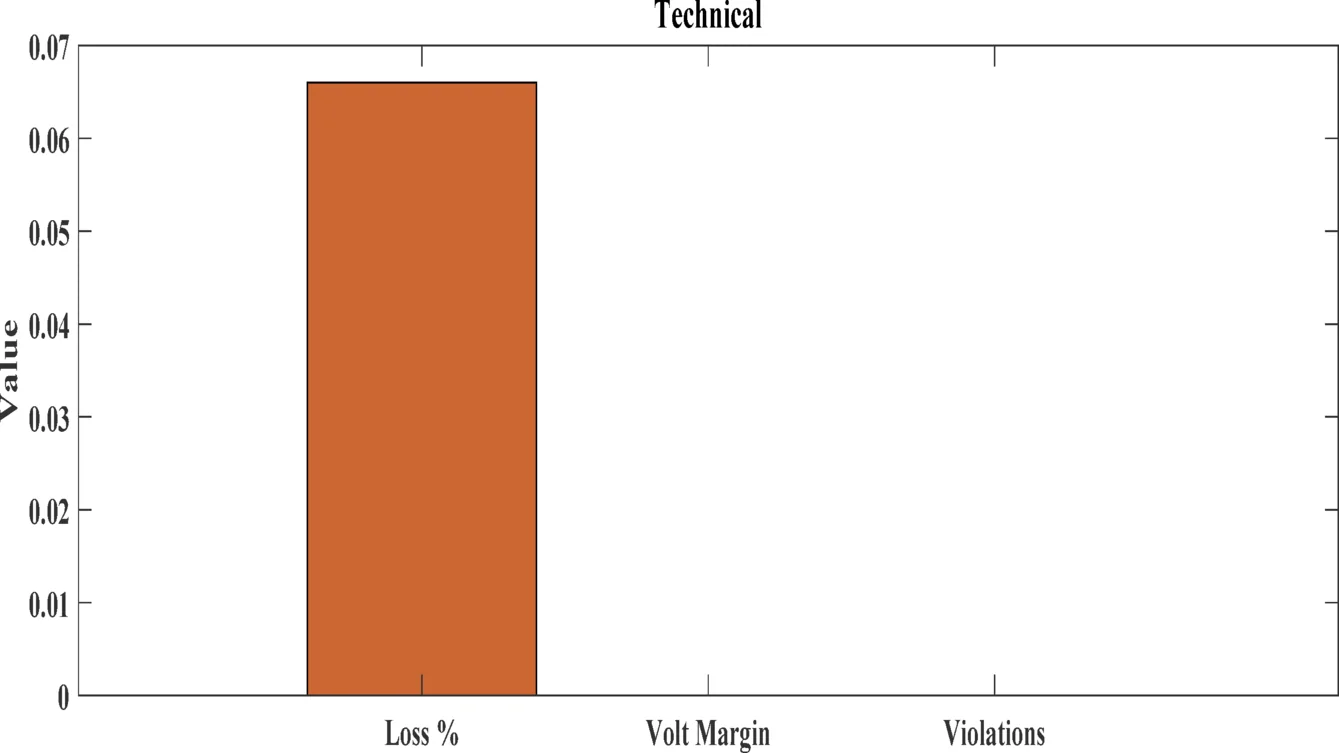
Technical performance.

**Fig. 24:**
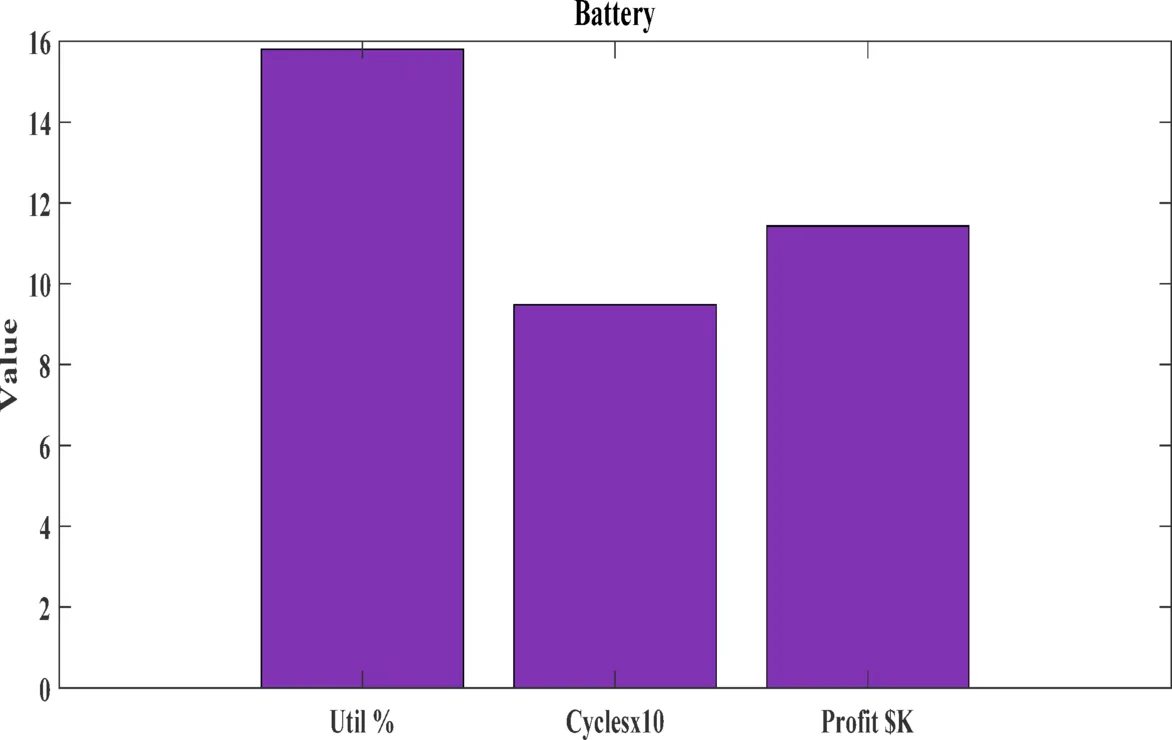
Battery performance indicators.

Figure 23 indicates a notable reduction in network losses (loss percentage 6%), with negligible voltage-margin violations, evidence of improved stability and effective DG placement. Figure 24 summarizes BESS performance in terms of utilization, cycling frequency, and profit: utilization is 16% with 1 full equivalent cycle per day, and profit is approximately $11k, consistent with price-arbitrage activity during peak periods.

## Environmental and economic KPIs

**Fig. 25:**
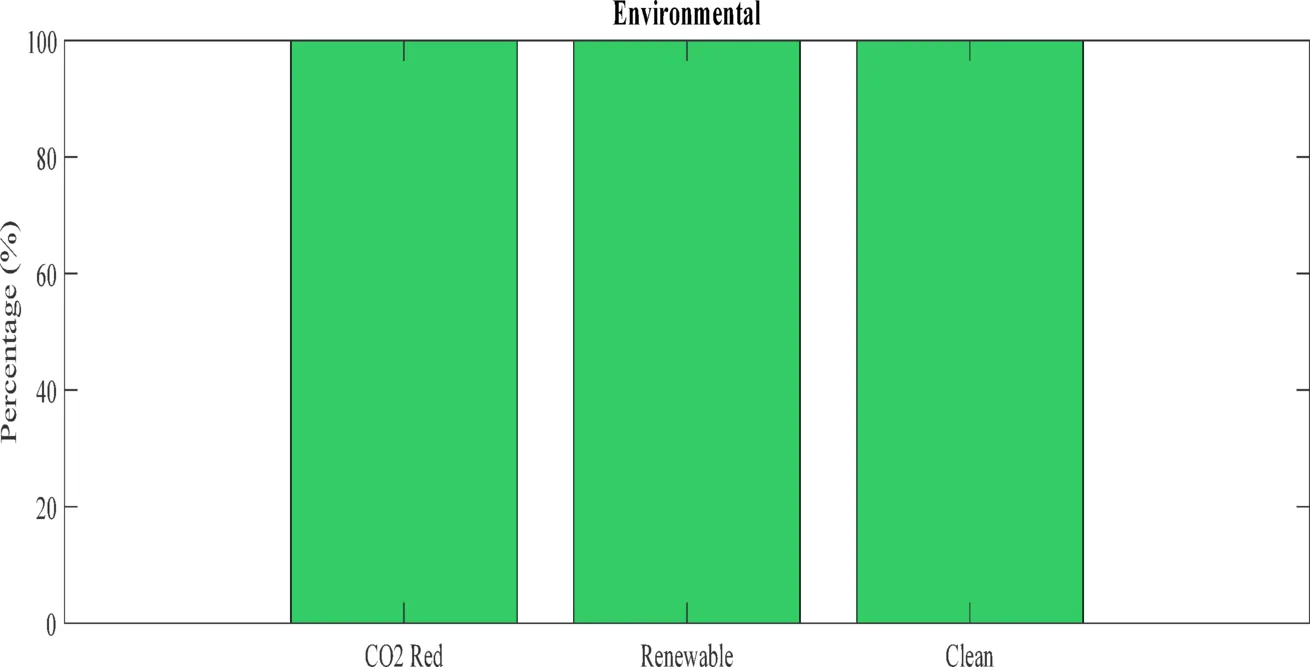
Environmental KPIs.

**Fig. 26:**
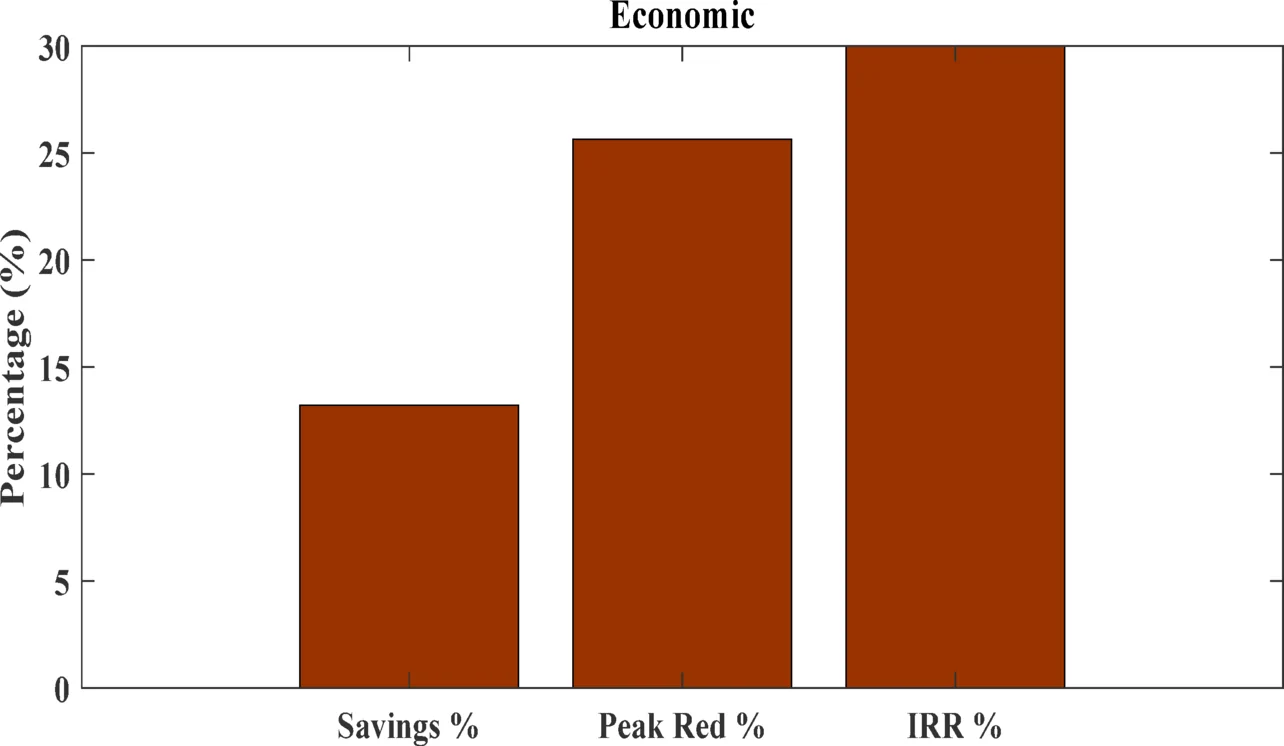
Economic KPIs.

Figure 25 shows uniformly strong environmental indicators (CO₂ reduction, renewable fraction, clean-energy utilization), reflecting the dominance of renewable resources within the VPP operating regime. Economic KPIs in Fig. 26 highlight substantial gains: daily savings exceed 13%, peak-demand reduction reaches 26%, and the internal rate of return approaches 30%, collectively validating the economic merit of coordinated DER and BESS operation under the VPP paradigm.

### Robustness, scalability, and practical implementation

The proposed NN-GWO framework demonstrates strong robustness and scalability for real-world VPP applications.

#### Scalability:

The framework can be scaled to larger distribution networks (e.g., IEEE 118-bus system) by adjusting the population size and iteration count of the GWO algorithm. The computational complexity scales linearly with the number of buses and DG units, making it suitable for systems with up to several hundred nodes.

#### Adaptability to Real-Time Operations:

The framework can be adapted for real-time operations by using the trained neural network for rapid load forecasting (prediction time < 1 s) and the GWO for periodic re-optimization (e.g., every 15 min). This enables a dynamic response to changing grid conditions.

#### Parameter Sensitivity:

The performance of the GWO algorithm is sensitive to population size and iteration count. Our analysis shows that population size = 30 and iterations = 100 provide an optimal balance between solution quality and computational time (approximately 45 s for the IEEE 69-bus system).

#### Practical Implementation:

The framework can be deployed in real-world VPP management systems using existing SCADA and EMS infrastructure. The neural network can be trained offline and deployed for online forecasting, while the GWO optimization can be integrated into the EMS for day-ahead and intra-day scheduling (see Table 7).

**Table 7:** Sensitivity analysis of key parameters.

Parameter	Values Tested	Best Value	Impact on Performance
**Population Size**	10, 20, 30, 50	30	Higher population improves solution quality but increases computation time
**Iterations**	50, 100, 200	100	More iterations improve convergence but with diminishing returns
**Forecasting Horizon**	1 h, 2 h, 6 h, 24 h	24 h	Longer horizon improves scheduling but reduces accuracy
**BESS Capacity**	0.5 MWh, 1 MWh, 2 MWh	2 MWh	Larger capacity improves flexibility but increases cost

### Strengths and limitations of the proposed approach

- **Strengths**:

1. Superior Convergence: The GWO algorithm demonstrates stable, non-oscillatory convergence, as shown in Fig. 5, with the best fitness value decreasing smoothly from approximately 0.482 to 0.4742 over 100 iterations.
2. High Forecasting Accuracy: The proposed NN-GWO framework achieves a mean prediction error of 1.27 MW, representing a 20.6% improvement over SVM and a 39.5% improvement over LSTM.
3. Multi-Objective Optimization: The framework successfully addresses technical (loss reduction, voltage profile), economic (cost savings, IRR, payback period), and environmental (CO₂ reduction) objectives simultaneously.
4. Practical Applicability: The framework can be deployed in real-world VPP management systems using existing SCADA and EMS infrastructure, with the neural network trained offline and deployed for online forecasting.
5. Scalability: The framework can be scaled to larger networks (e.g., IEEE 118-bus system) by adjusting the population size and iteration count of the GWO algorithm.

- **Limitations**:

1. Computational Time: The computational time increases with network size, approximately 45 s for the IEEE 69-bus system, scaling to several minutes for larger networks with more than 100 buses.
2. Parameter Sensitivity: The performance of the GWO algorithm is sensitive to key parameters such as population size and iteration count. Our analysis identified population size = 30 and iterations = 100 as providing an optimal balance.
3. Forecasting Dependency: The scheduling accuracy depends on the quality of the forecasting model. Under extreme weather conditions (e.g., sudden cloud cover, unexpected wind gusts), PV and WT generation forecasts may deviate, potentially affecting optimal scheduling.
4. Extreme Conditions: Performance degrades under extreme weather conditions affecting PV and WT generation, highlighting the need for robust uncertainty handling mechanisms.

- **Computational Characteristics**:
- Average computation time: 45 s for the IEEE 69-bus system.
- Population size: 30, Iterations: 100 provide optimal balance.
- Memory requirement: approximately 2 GB for the full framework.

## Conclusion

This study makes three key contributions to the field of VPP management: (1) the development of a hybrid NN-GWO optimization methodology for simultaneous load forecasting and DG scheduling, (2) a multi-objective formulation addressing technical, economic, and environmental performance, and (3) rigorous validation on a realistic radial distribution network demonstrating significant improvements in loss reduction, voltage profile, cost savings, and emissions reduction.

In order to improve the performance of the IEEE 69-bus distribution system, this study proposed an integrated Virtual Power Plant (VPP) framework that combines optimally sized Distributed Generation (DG) units, renewable energy resources, machine learning-based load forecasting, and a coordinated. The best locations and capacities of DG units were found using the Grey Wolf Optimizer (GWO), which significantly improved network technical performance. By increasing the minimum bus voltage from 0.909 p.u. to 0.966 p.u. and reducing real power losses by 61.74%, the optimized configuration showed how judicious DG placement may strengthen weak network nodes and mitigate voltage drops. Reliable daily demand profile projections were made possible by the suggested machine learning-based forecasting model, which allowed for effective operational scheduling of energy storage and renewable generation resources. The BESS, wind power, and photovoltaic (PV) systems worked together to provide about 4.2 MWh of renewable energy each day, successfully meeting system demand during crucial times. By charging during off-peak hours and discharging during high-demand times, the BESS also executed sophisticated energy shifting, increasing system flexibility and optimizing energy use. The advantages of the suggested VPP framework over a traditional grid-dependent operation were further validated by the economic analysis. With a discounted payback period of 3.8 years, an internal rate of return (IRR) of 30%, and daily operational cost reductions of $1,741.32, the system demonstrated high economic viability. Increased renewable penetration dramatically decreased CO2 emissions from an environmental standpoint, demonstrating the potential of VPP-based systems to facilitate sustainable energy transitions. Overall, the findings show that in contemporary distribution networks, the coordinated integration of distributed generation, renewable energy resources, and energy storage within a single VPP architecture significantly improves technical performance, lowers operating costs, and enhances environmental sustainability. To further improve the effectiveness, flexibility, and robustness of intelligent VPP systems, future research topics can include the creation of sophisticated multi-objective optimization frameworks, real-time coordinated control techniques, and market-oriented dispatch mechanisms.

## Data Availability

All data generated or analyzed during this study are included in this article.

## Abbreviations

RERs: Renewable Energy Resources
VPP: Virtual Power Plant
EP: Electricity Pricing
RE: Renewable Energy
ATC: Aggregated Technical and Commercial Losses
DR: Demand Response
DERs: Distributed Energy Resources
EVs: Electric Vehicles
DA: Day-Ahead
RT: Real-Time
MG: Microgrid
KPIs: Key Performance Indicators
GJO: Golden Jackal Optimization
PVD: Peak-to-Valley Differences
DG: Distributed Generation
EV: Electric Vehicle
GT: Gas Turbine
PV: Photovoltaic
WP: Wind Power
VD: Voltage Deviation
C&CG: Column-and-Constraint Generation
LB / UB: Lower Bound / Upper Bound
RPSC: Remaining Peak Shaving Capability
ML: Machine Learning
ω₁ / ω₂: Weights for peak shaving/voltage regulation
Fₚvd / F_v_ₐ_r_: Net load peak-to-valley difference/fluctuation variance
a / b: Weights of Fₚvd and F_v_ₐ_r_
N: Set of all nodes in the distribution network
V_i_: Voltage at node i
V_i_ᵐᵃˣ / V_i_ᵐᶦⁿ: Upper / lower voltage limits at node i
V_r_ₑf_i_: Rated voltage at node i
Cₚₖ / C_r__v_: Compensation coefficients for peak shaving / voltage regulation
α / β: Standards for net-load PVD / fluctuation
λ: Standard for voltage deviation
VPP: Virtual Power Plant
DG: Distributed Generation
BESS: Battery Energy Storage System
PV: Photovoltaic
WT: Wind Turbine
GWO: Grey Wolf Optimizer
ANN: Artificial Neural Network
NN: Neural Network
ML: Machine Learning
SVM: Support Vector Machine
RES: Renewable Energy Sources
DERs: Distributed Energy Resources
P_loss: Active power losses
V_i: Voltage at bus i
SOC: State of Charge
IRR: Internal Rate of Return
NPV: Net Present Value
